# Confirmation of the Disulfide Connectivity and Strategies for Chemical Synthesis of the Four-Disulfide-Bond-Stabilized *Aspergillus giganteus* Antifungal Protein, AFP

**DOI:** 10.1021/acs.jnatprod.2c00954

**Published:** 2023-02-27

**Authors:** Györgyi Váradi, Gyula Batta, László Galgóczy, Dorottya Hajdu, Ádám Fizil, András Czajlik, Máté Virágh, Zoltán Kele, Vera Meyer, Sascha Jung, Florentine Marx, Gábor K. Tóth

**Affiliations:** † Department of Medical Chemistry, University of Szeged, Szeged 6720, Hungary; ‡ Department of Organic Chemistry, University of Debrecen, Debrecen 4010, Hungary; § Institute of Molecular Biology, Biocenter, Medical University of Innsbruck, Innsbruck 6020, Austria; ∥ Institute of Biochemistry, Biological Research Centre, Eötvös Loránd Research Network, Szeged 6726, Hungary; ⊥ Department of Applied and Molecular Microbiology Technische Universität Berlin, Institute of Biotechnology, Berlin 13355, Germany; # MTA-SZTE Biomimetic Systems Research Group, University of Szeged, Szeged 6720, Hungary; ○ Department of Microbiology, Faculty of Science and Informatics, University of Szeged, Szeged 6726, Hungary

## Abstract

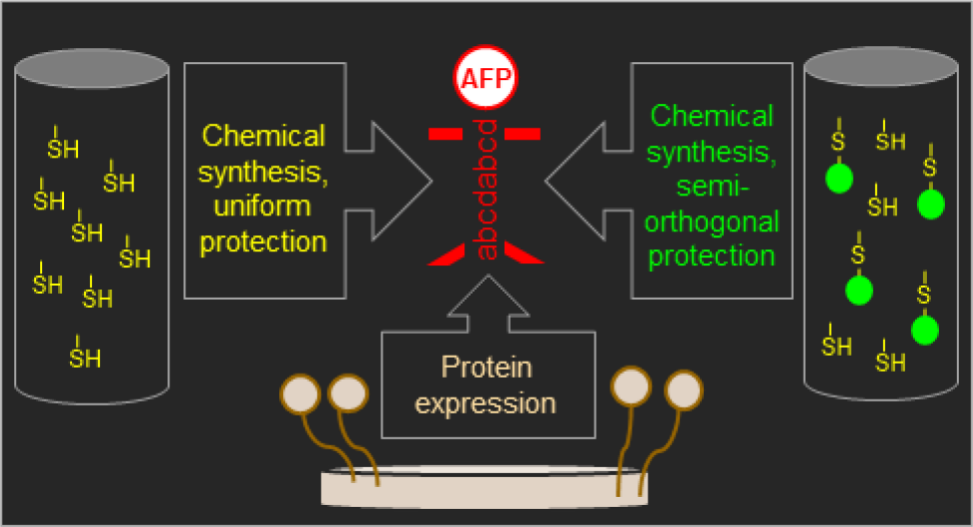

Emerging fungal infections require new, more efficient antifungal agents and therapies. AFP, a protein from *Aspergillus giganteus* with four disulfide bonds, is a promising candidate because it selectively inhibits the growth of filamentous fungi. In this work, the reduced form of AFP was prepared using native chemical ligation. The native protein was synthesized via oxidative folding with uniform protection for cysteine thiols. AFP’s biological activity depends heavily on the pattern of natural disulfide bonds. Enzymatic digestion and MS analysis provide proof for interlocking disulfide topology (*abcdabcd*) that was previously assumed. With this knowledge, a semi-orthogonal thiol protection method was designed. By following this strategy, out of a possible 105, only 6 disulfide isomers formed and 1 of them proved to be identical with the native protein. This approach allows the synthesis of analogs for examining structure–activity relationships and, thus, preparing AFP variants with higher antifungal activity.

Due to the rapid increase in the number of fungal infections, antifungal proteins are of particular interest.^1^ The *Aspergillus giganteus* antifungal protein (AFP), a cysteine-rich antifungal protein from certain Ascomycetes, was first isolated and characterized in 1965.^2^ AFP is a promising biotechnological antifungal compound used to fight against filamentous fungi.^3^ Until today, several members of this protein group were described from taxonomically distinct species, such as *Aspergillus* spp., *Fusarium* spp. *Penicillium* spp., and *Monascus* spp. The presence of hypothetical orthologues is supposed in several other filamentous fungi.^4−9^ Due to a high amount of arginine and lysine residues, this protein group has some common features such as low molecular mass and a cationic character.^10^ Although the secreted, mature proteins differ in their amino acid sequences, they have similar predicted protein folding patterns: five antiparallel β-strands linked by loops with a β-barrel topology.^7,11−13^ Using NMR spectroscopy, this structural property was confirmed experimentally in the cases of AFP,^11^ the *Penicillium chrysogenum* antifungal proteins (PAF,^12^ PAFB,^14^ and PAFC^15^), and NFAP from *Neosartorya fischeri*.^16^ Intramolecular disulfide bonds between cysteine residues, which provide high stability against protease degradation at high temperatures and within a broad pH range, stabilize the compact protein structure.^10^ The six cysteines of PAF, PAFB, and NFAP form three disulfide bonds in the *abcabc* pattern,^12,14,16,17^ while in AFP and PAFC, four disulfide bridges connect the eight cysteines.^11,15^ The AFP solution structure was determined using NMR.^11^ The presence of all disulfide bonds and the formation of the correct pattern proved essential for structural integrity and antifungal activity.^18^ Previously contradictory results came from the disulfide bond pattern of AFP by assuming the existence of minor components having unnatural disulfide bridges.^11^ According to a recent study, the interlocking *abcdabcd* disulfide bonding is the most probable for AFP (Figure 1).^19^ However, the actual disulfide bond pattern remains unclear. It is worth noting that the presence of two cysteines separated by a single amino acid at the C-terminus of AFP complicates mapping disulfide bonds.

**Figure 1:**
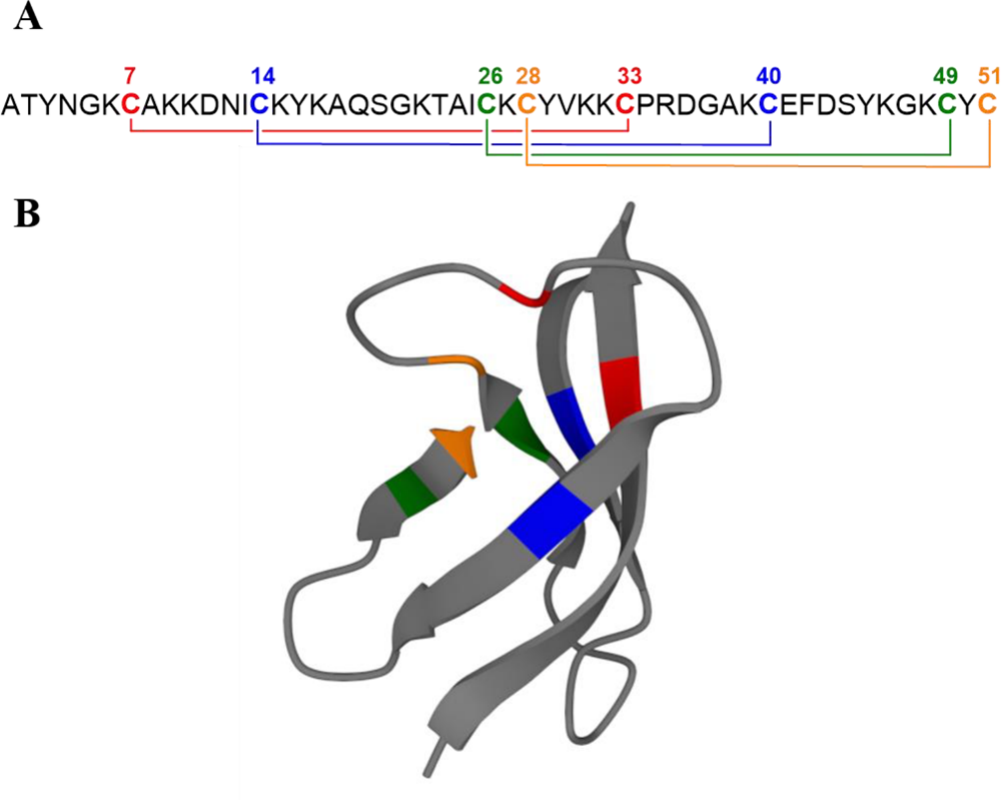
Disulfide bond pattern of AFP (*abcdabcd*) that seemed most likely in previous studies (A).^11,19^ Structure of AFP (pdb code 1AFP) highlighting the cysteines with colors. (B) The colors represent the same cysteines in both figures, and the coloring corresponds to the most likely disulfide pattern.

Although wild-type proteins can be produced by recombinant expression,^12,14,15,20−22^ preparation of non-natural protein analogs to increase efficiency or to investigate structure–activity relationships is not always feasible by biological methods. Chemical synthesis may remedy improper protein processing. However, the formation of designed disulfide bonds by chemical methods is still challenging. The endoplasmic reticulum of eukaryotic cells is the site of oxidative protein folding, supported by many chaperones and cofactors.^23,24^ Enzymes of the protein disulfide isomerase (PDI) family can rearrange incorrectly formed disulfide bridges by oxidizing cysteine thiols and reducing disulfide bonds.^25−27^ Apart from these well-organized biological processes, oxidation by chemical reagents can produce a variety of disulfide isoforms. The correct disulfide bridge pattern is essential for many proteins’ structural integrity and biological activity. Therefore, it is important to prevent disulfide isomer formation. As the number of cysteines increases, the number of possible disulfide bond variants increases rapidly. Proteins with four cysteines can give rise to only three possible isomers, but with six cysteines, there are 15, and with eight cysteines, there are 105 possible disulfide bridge patterns.

The AFP-related antifungal protein PAF was successfully synthesized by uniformly protecting the cysteine sulfhydryl groups.^17^ Folding applying both an oxidizing and a reducing agent led to the native *abcabc* pattern proved by enzymatic digestion and mass spectrometry (MS). This strategy could also apply to the chemical synthesis of AFP based on the structural similarities of PAF and AFP. However, AFP contains eight cysteines, which can complicate the correct pairing of the cysteine thiols. The outcome of the chemical synthesis approach is more questionable if a new protein analog is synthesized instead of the parent protein. Generally, a protein with the native disulfide bond pattern is supposed to adopt the thermodynamically most stable conformation.^28^ In contrast, protein analogs might adopt conformations and disulfide patterns according to transient thermodynamic energy minima which lie above the native state. Selective protection of the cysteine side chains may provide a solution to this problem.^29−32^ However, finding suitable orthogonal protecting groups for four pairs of cysteines is difficult. A thorough literature search concludes that peptides and proteins prepared by selective thiol protection contain a maximum of three disulfide bonds. A recent review provides a single example of orthogonal protection of four cysteine pairs. This strategy can be used only if the peptide or protein is synthesized by 9-fluorenylmethyloxycarbonyl (Fmoc) chemistry.^33^ This is usually not a problem, but when native chemical ligation (NCL) must be applied for the generation of the full-length protein, *tert*-butyloxycarbonyl (Boc) chemistry avoiding the use of a base is a better choice for the thioester-containing segment.

This paper reports the chemical synthesis and determination of native AFP’s disulfide connectivity and a synthetic strategy using semi-orthogonal thiol protecting groups for preparing AFP analogs possessing native disulfide bond patterns. These synthetic strategies allow the generation of modified antifungal proteins to study the structure–function relationship, which paves the way for producing AFP variants with even improved antifungal efficacy.

## Results and Discussion

### Stepwise Synthesis of AFP

Stepwise solid-phase synthesis is the most basic and popular technique for preparing peptides or proteins. The mature AFP is a 51-mer protein. Because the upper limit of stepwise synthesis is approximately 50 amino acids, AFP is at the border between those proteins that can be prepared by stepwise synthesis and those that cannot.^34^ The preparation of AFP failed despite the use of a very effective technique, microwave-assisted solid-phase peptide synthesis (Figures S1 and S2). Despite attempts to use more efficient coupling agents and optimize the reaction conditions, the desired protein could not be obtained. In addition to the protein’s length, the failure is attributable to “difficult sequences”.^35^ These are regions of peptides or proteins prone to the formation of β-sheet secondary structures and thus aggregation during solid-phase synthesis. Aggregation potential (*P*_a_) for the residues was determined with the peptide calculator of CEM Corporation (https://cem.com/en/peptide-calculator), which is based on the measurement of the resin’s swelling capacity.^35^ If the *P*_a_ of a residue is greater than 1.1, it has a strong propensity to aggregate. *P*_a_ of two amino acids exceeds 1.1 in the critical region of 6–9 carboxy-terminal residues, strongly impacting the whole sequence. Between Gly21 and Val30, the aggregation potentials of 10 consecutive amino acids are greater than 1.1. After five amino acids, there is a seven-residue-long region with *P*_a_ above 1.1. Altogether, 45% of the amino acids in AFP have an aggregation potential of 1.1 or more. The length, together with the aggregation profile of AFP, may be responsible for unsuccessful stepwise synthesis.

### Synthesis of AFP by NCL

Due to the failure of stepwise synthesis and considering the presence of cysteines in the protein, NCL seemed to be the most suitable method to prepare AFP. The preferred technique for the homologous PAF was discovered to be this significant extension of peptide synthesis.^17^ As one of the eight cysteines (Cys^28^) is located in the middle of AFP, the N-terminal 1–27 and the C-terminal 28–51 fragments seemed appropriate for ligation. The thioester of the N-terminal part, the crucial component of chemical ligation, was synthesized on the previously published Cys-SH resin applying Boc/benzyl (Bzl) chemistry (Figures S3, S4, and S19).^17^ The advantage of this method is that it works well for any C-terminal amino acid. Furthermore, it provides a thioester that is reactive enough to undergo chemical ligation in a relatively short reaction time. The C-terminal fragment was synthesized in solid-phase using Fmoc/*tert*-butyl (*t*Bu) chemistry (Figures S5, S6, and S20). NCL of the purified fragments was carried out in a 0.1 M ammonium acetate (NH_4_OAc) buffer (pH 7.5) containing 3% (w/v) thiophenol as a thiol adduct. The reaction was completed within 3–4 h in all cases (Figure S7). The average yield was 35%.

### Disulfide Bond Formation of Uniformly Protected Cysteines

**Scheme 1:**
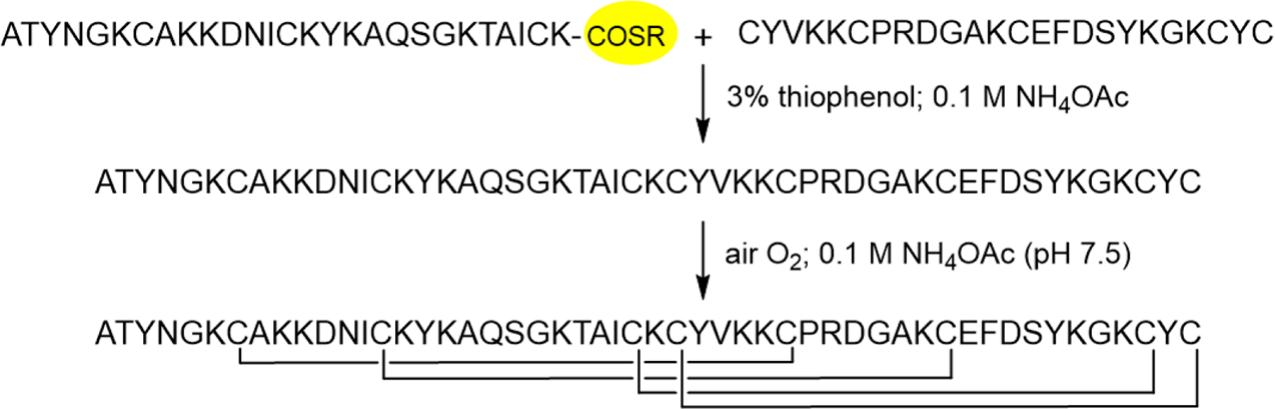
Synthesis of AFP by Uniform Protection of Cysteines

In the first strategy aimed at synthesizing native AFP, uniformly side-chain protected cysteine residues were built into the protein (Scheme 1). In the N-terminal thioester fragment synthesized by Boc chemistry, the thiol protecting group was 4-methylbenzyl (Meb). In contrast, in the C-terminal part prepared by Fmoc chemistry, sulfhydryl groups were protected by trityl (Trt). Cleavage of the peptides from the resins and concomitant removal of side-chain protecting groups followed by NCL of the fragments generated eight free thiols to be oxidized (Figures S8 and S21). Although the presence of a reducing agent such as cysteine or reduced glutathione in the oxidizing mixture was found to be essential for the folding of PAF,^11^ it promoted the generation of many different disulfide isoforms of AFP (Figure S9). Thus, instead of a redox mixture, only an oxidizing agent, namely molecular oxygen was applied in this case. The reduced form of AFP was dissolved in NH_4_OAc buffer (pH 7.5) at a 0.2 mg mL^–1^ concentration to avoid the formation of intermolecular disulfide bonds, and air oxygen was stirred intensively into the solution at room temperature (Figures S10 and S11).

**Figure 2:**
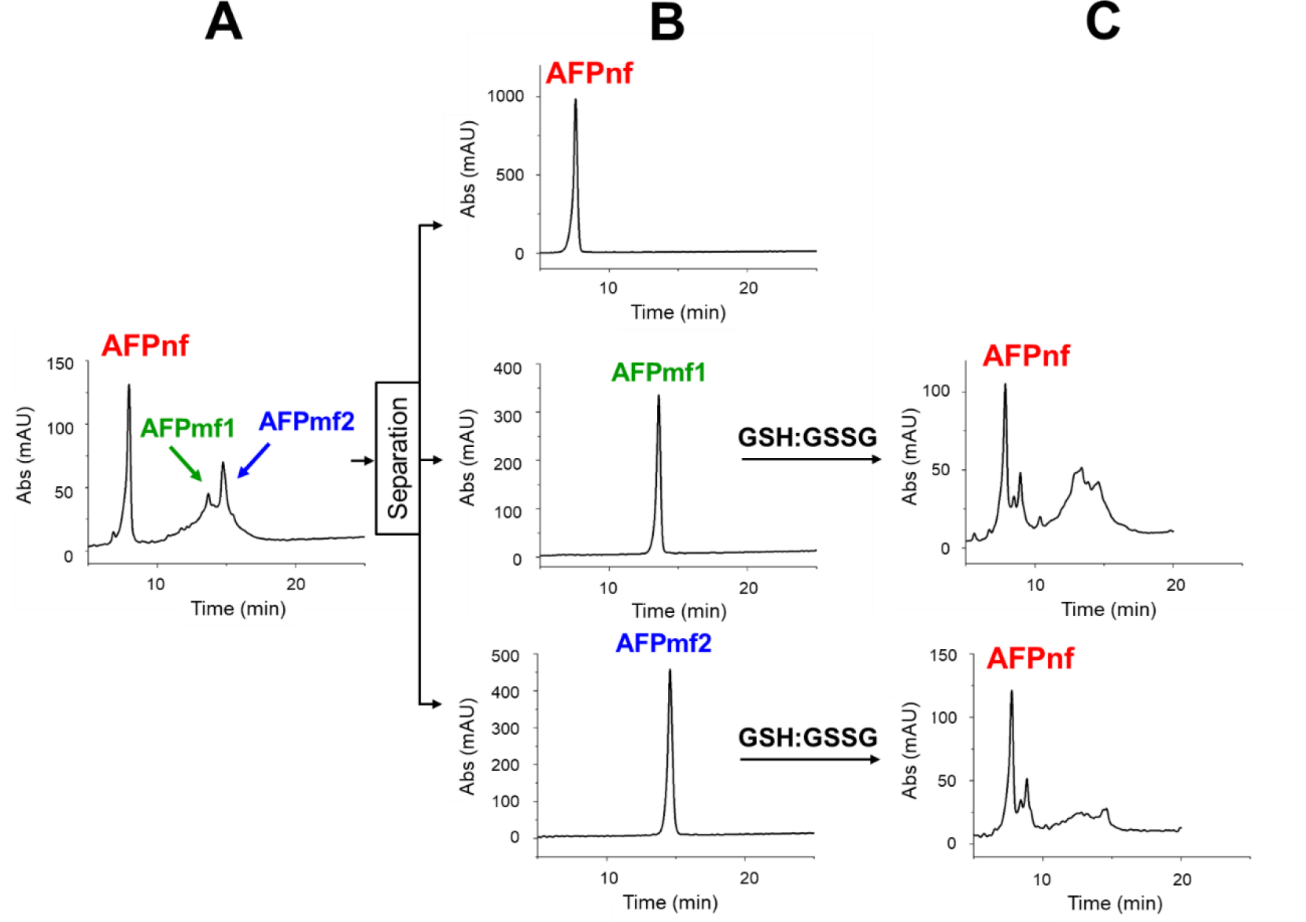
Folding of AFP synthesized by uniform protection of cysteine thiols. Air oxidation resulted in three disulfide isomers (A), which were separated by RP-HPLC (B). The least hydrophobic isomer (AFPnf) was proved to have natural disulfide connectivity. The misfolded isoforms (AFPmf1 and AFPmf2) could be refolded to the protein containing the native disulfide bond pattern by treatment with a glutathione redox system (C).

Out of the 105 possible disulfide isomers, only one major and two minor products were formed (Figures S12 and 2A). The isoforms were separated by reversed-phase high-performance liquid chromatography (RP-HPLC). The major natural form product (AFPnf) eluted at a shorter retention time in RP-HPLC, suggesting that it was the least hydrophobic component. This protein was proved to be identical to native AFP (AFPN). Both minor products (AFPmf1 and AFPmf2) were treated with a glutathione redox system having a GSH:GSSG ratio of 5:1, and their disulfide bonds were reshuffled to the native pattern spontaneously (Figure 2). The glutathione redox system has the same role as enzymes of the PDI family. Namely, it can oxidize thiols to disulfide bridges and reduce incorrectly formed disulfides to thiols.^36^ The process is likely under thermodynamic control, where unstable isoforms are converted to more stable ones.

### NMR Investigations

**Figure 3:**
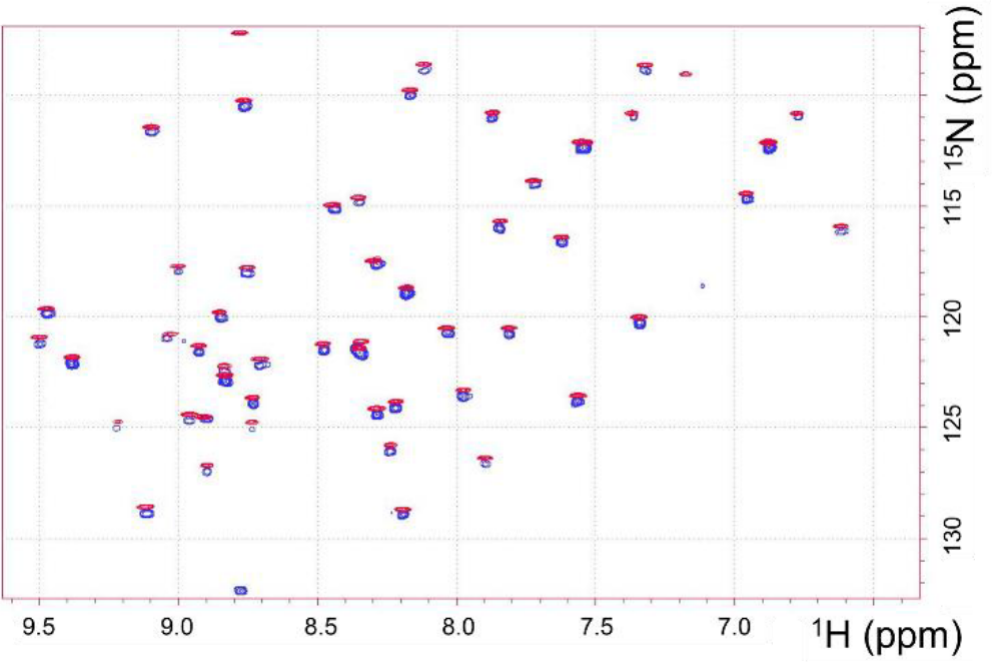
Comparison of the ^1^H–^15^N HSQC spectra of AFPs: ^15^N-labeled AFP (AFPN) (red) and synthetic AFP (AFPnf) (blue). The two spectra are slightly shifted for better visibility.

**Figure 4:**
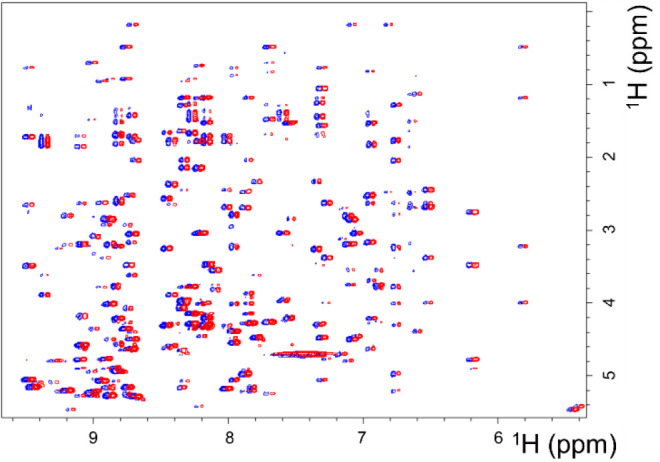
Comparison of the partial ^1^H–^1^H NOESY spectra of AFPs: ^15^N-labeled AFP (AFPN) (red) and synthetic AFP (AFPnf) (blue). ^15^N-Decoupling was applied during acquisition for the ^15^N-AFP sample to remove signal splitting. The two spectra are slightly shifted for better visibility.

The synthetic AFP (AFPnf) and authentic native AFP (AFPN) were found to be identical according to NMR standards, as demonstrated by the identity of ^1^H (Figure S25), ^1^H–^15^N HSQC (Figure 3), and ^1^H–^13^C HSQC spectra (Figure S26) and even by the ^1^H–^1^H NOESY spectrum (Figure 4).

### Identification of Disulfide Bond Pattern by MS

Mass spectrometric approaches for disulfide-bridge identification are based on chemical and enzymatic methods (according to the protein sequence or peptide sequence) and produce a mixture of peptides containing only one disulfide bond. Capillary RP-HPLC coupled to the mass spectrometer is used to separate and analyze fragments linked by disulfide bridges. The fragments can be identified based on their unique masses and tandem MS fragments. The reagents employed to cleave the peptide or protein at the half-cysteinyl residues determine whether the operation is successful. A mixture of trypsin and chymotrypsin seemed to be a good choice based on the AFP sequence. With this enzyme cocktail, there would be at least one cleavage site between any two neighboring cysteine residues (trypsin cleaves at the N-terminus of K and R; chymotrypsin cleaves at the N-terminus of F, W, and Y). Hence MS analysis of the digestion mixture might be used to identify disulfide-linked fragments. However, cleavage of AFP by trypsin–chymotrypsin mixture would lead to the formation of the CY dipeptide twice (positions 7, 8, and 49, 50), which would make the analysis ambiguous. Trypsin alone can cleave the protein between any two neighboring cysteines except Cys49 and Cys51 of CYC, so pairs of peptide fragments linked together by disulfide bridges can be obtained for analysis. The CYC peptide can bind to two other peptides because it contains two cysteines; consequently, the exact positions of disulfide bridges cannot be determined by MS analysis alone. A fragmentation spectrum of the tripeptide would also be required. For these reasons, only trypsin was used for enzymatic cleavage of AFP, and all putative disulfide-bridged fragments were subjected to MS/MS analysis.

MS and software analysis analyzed the first 50 most intense peaks in the tryptic digest of AFP. The pair of peptide fragments DNICK-CEFDSYK having a disulfide bond between cysteines 14 and 40 was identified based on its mass (494.2079^3+^ ion measured with 1 ppm accuracy), and MS/MS confirmed its structure. In the MS/MS spectrum, both peptides were visible independently (at nominal masses 592 and 891 due to fragmentation of the disulfide bond), and an almost complete C-terminal (y) ion series of the CEFDSYK peptide could be observed (Figure S27). Another pair of peptide fragments possessing disulfide linkage between cysteines 7 and 33 and having 457.2225^2+^ nominal mass ion (measured with 0.5 ppm accuracy) was also identified. The measured mass was presumably referred to as the CYK-KCPR structure. MS/MS spectrum showed fragment peaks corresponding to the supposed peptide (Figure S28).

Due to the lack of a trypsin digestion site between cysteines 49 and 51, a disulfide-linked triplet was expected and found (477.2042^3+^ mass measured with 0.06 ppm accuracy). Analysis of ions above 800 Da in the spectrum revealed that it was CYC coupled to TAICK and CYVK. Disulfide connectivity of the triplet (CYVK-CYC-TAICK or TAICK-CYC-CYVK) was determined by fragmentation of the CYC part and MS/MS analysis. During fragmentation of an amide bond in the higher-energy collisional dissociation cell of MS, mostly b- and y-type ions can form. The b ion originates from the N-terminal part of the peptide, and its mass is equal to the sum of the residue masses of the peptide. The y ion derives from the C-terminal part of the peptide, and its mass is equal to the mass of the peptide calculated from the sequence (Figure S29, A). The mass difference between fragments YC-CYVK and CYVK-CY is 18 Da. Therefore, ions with nominal masses of 794 and 631 belong to the C-terminal part of the peptide. Thus, CYVK (containing Cys28) is connected to Cys51; consequently, Cys49 is linked to TAICK enclosing Cys26 (Figure S29, B). These results verify the presence of a disulfide bond between cysteines 26 and 49 and another between cysteines 28 and 51. The disulfide bond pattern of AFP was *abcdabcd*.

### Antifungal Activity Assay

**Figure 5:**
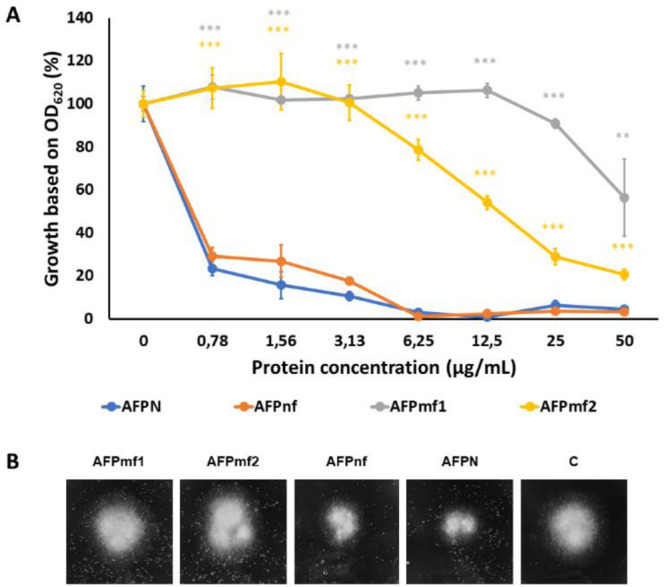
Growth inhibition potential of AFPN, AFPnf, AFPmf1, and AFPmf2 against *Aspergillus niger* SZMC 601 in (A) broth microdilution assay and (B) on surface colonies after incubation for 48 h at 25 °C in the presence of (A) 0–50 μg mL^–1^, and (B) 1 μg mL^–1^ protein. (A): The untreated control culture (0 μg mL^–1^ protein) was considered as 100% growth. *** (*p* < 0.0001) and **p (<0.005) indicate the significant difference in the growth values (mean ± standard deviation) of samples in comparison with the AFPN-treated sample at the respective concentrations. AFPN: native *Aspergillus giganteus* antifungal protein, AFPnf: synthetic *A. giganteus* antifungal protein with native disulfide bond pattern, AFPmf1, and AFPmf2: misfolded synthetic *A. giganteus* antifungal proteins, C: untreated control. The untreated control was set to represent 100% growth.

*Aspergillus niger* was used to examine the biological activity of native and synthetic AFP in a broth microdilution assay because it is an excellent model organism to study the antifungal activity of AFP on filamentous fungi.^36^ AFPN and AFPnf were similarly active and inhibited the growth of *A. niger* at a minimal inhibitory concentration (MIC) of 6.25 μg mL^–1^. The misfolded isoforms (AFPmf1 and AFPmf2) were less effective. AFPmf1 was technically inactive, while AFPmf2 inhibited the growth of *A. niger* by only 21 ± 5% when used at the MIC of AFP/AFPnf, (Figure 5A). Overall growth inhibition was not detected even at the maximum used concentration (50 μg mL^–1^) of AFPmf1 and AFPmf2 (Figure 5A). AFPN and AFPnf reduced the colony diameter of *A. niger* surface colonies grown on a solid medium when they were used at 1 μg mL^–1^, while the misfolded isoforms AFPmf1 and AFPmf2 showed no inhibitory effect under these cultivation conditions (Figure 5B). Previous studies showed that the presence of all disulfide bridges was important for structural integrity and antifungal activity of AFP^18^ and the AFP-related *P. chrysogenum* PAF.^15^ Our data from antifungal activity assays highlighted the significance of the correct disulfide bond pattern for the entire antifungal activity, which has not been shown for any cysteine-rich antifungal protein from ascomycetes before.

### Disulfide Bond Formation of Semi-orthogonally Protected Cysteines

**Scheme 2:**
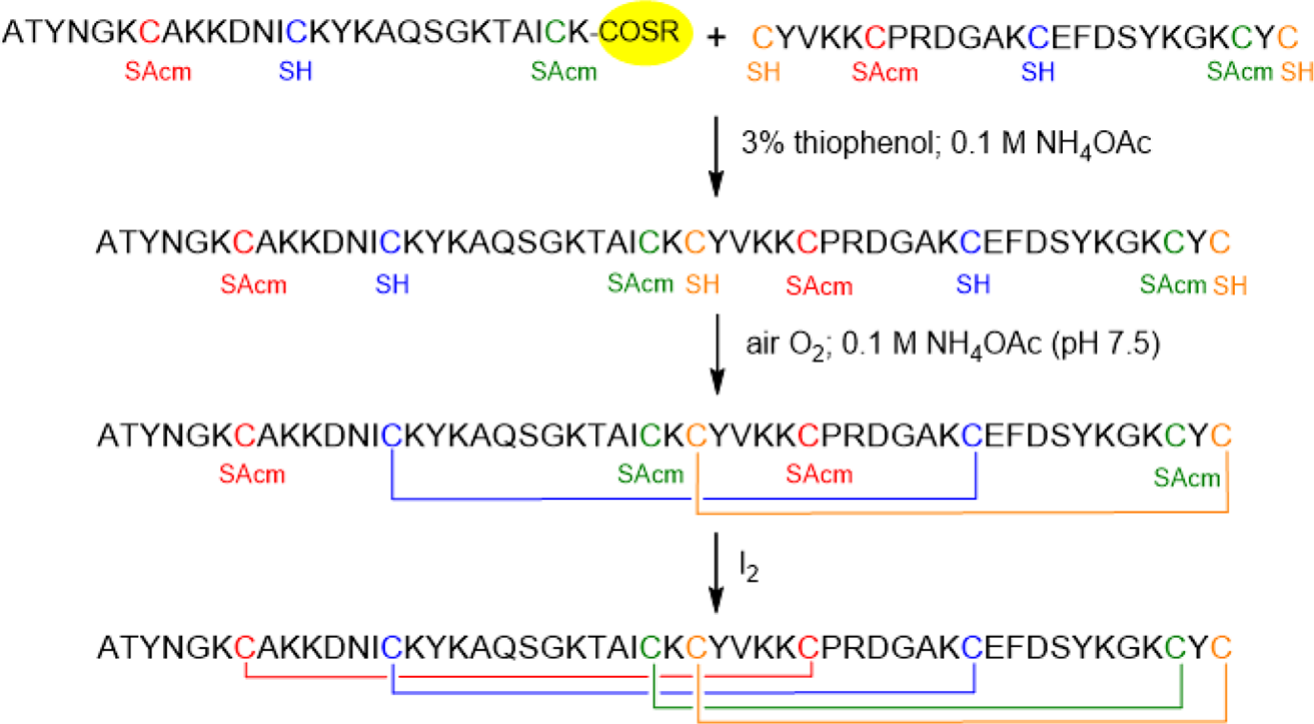
Synthesis of AFP by Semi-orthogonal Protection of Cysteines

**Figure 6:**
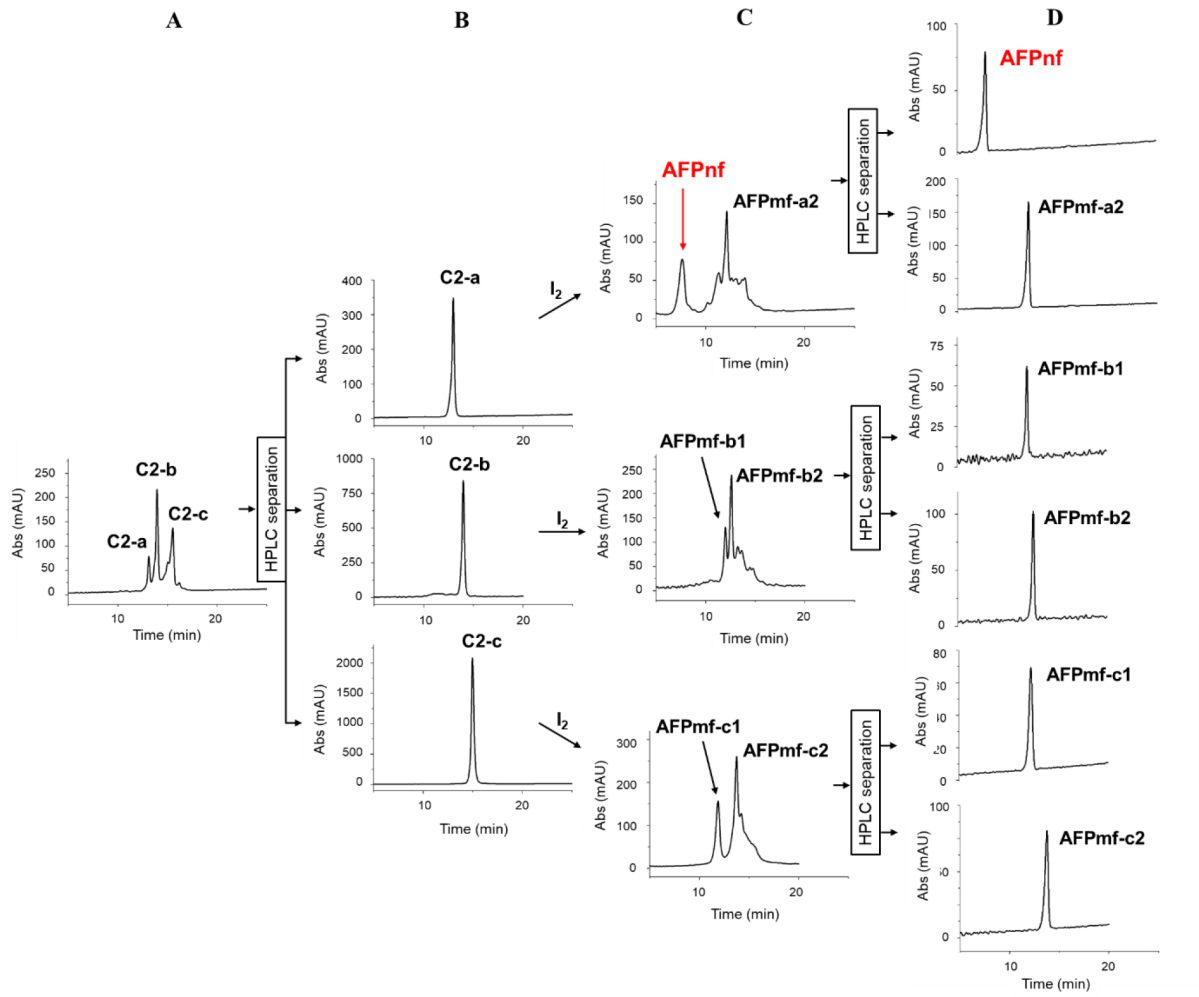
Folding of AFP synthesized by semi-orthogonal protection of cysteine thiols. AFP(Acm_2_)-C2 isomers (globular, bead and ribbon, C2-a, C2-b, and C2-c, respectively) (A) were separated by RP-HPLC (B) and treated with iodine (C). The naturally folded protein (AFPnf) and five misfolded disulfide isomers were isolated (D).

Natural cysteine pairing may not result from spontaneous folding under oxidative conditions if a native protein or its analog is not thermodynamically stable. Regioselective disulfide bond formation is possible in this situation. A semi-orthogonal protection strategy was used because there are not sufficiently different and suitable protecting groups for four disulfide bridges compatible with Boc and Fmoc chemistry (Scheme 2). Four cysteines (Cys14, Cys 28, Cys 40, and Cys51) were protected by a group that is cleaved simultaneously with the detachment of the protein from the resin, namely Meb and Trt in Boc and Fmoc synthesis, respectively, and the other four (Cys7, Cys26, Cys33, and Cys49) were protected by acetamidomethyl (Acm). Acm is a good choice because it remains intact during the cleavage of the peptide or protein from the resin in both Boc and Fmoc synthesis and during NCL. (RP-HPLC profiles and mass spectra of the two protein fragments are displayed in Figures S13–S16, S22, and S23.) First, we planned to form the disulfide bridges Cys14-Cys40 and Cys28-Cys51 because the former connects two of the five β-sheets, thereby stabilizing the β-barrel topology, and the latter connects the C-terminal to the center of the protein, thus further increasing the stability. Following NCL (Figures S17, S18, and S24), the four free thiols were oxidized by air oxygen. Peptides or proteins containing four cysteines can form three possible disulfide bridges: globular (Cys1-Cys3 and Cys2-Cys4), ribbon (Cys1-Cys4 and Cys2-Cys3), and bead (Cys1-Cys2 and Cys3-Cys4).^19^ As shown in Figure 6A, all three AFP(Acm_2_)-C2 isomers appeared in the reaction mixture. After separation by RP-HPLC, the three proteins were digested with trypsin (Figure 6B). MS measurements were performed in automated data-dependent acquisition (DDA) mode to determine their disulfide patterns. The least hydrophobic protein (C2-a) was globular, considering the first 20 most intense peaks, C2-b a bead, and the most hydrophobic (C2-c) a ribbon isomer. All three were treated with iodine to remove Acm groups and oxidize thiols to disulfide bonds.

Even though theoretically three isomers could have been formed from the four free sulfhydryl groups, only two main isomers were detected in all three cases (Figure 6C). Six different AFP disulfide isoforms were obtained (Figure 6D). First, the applied semi-orthogonal protection strategy could decrease the number of disulfide isomers from the theoretical 105 to only 6. Second, the least hydrophobic one (AFPnf) proved identical to the native protein (AFPN).

## Conclusions

AFP from *A. giganteus* belongs to a unique class of antifungal proteins secreted by certain filamentous ascomycetes.^9^ Four disulfide bonds that the protein’s eight cysteines create are essential for structural stability and antifungal activity. Enzymatic cleavage and MS analysis of the fragments demonstrated the interlocking disulfide linkage pattern (*abcdabcd*).

Presumably, due to the length of AFP (51-mer) and the presence of “difficult sequences” (fragments tend to aggregate), the synthesis of the full-length protein was not feasible by stepwise condensation. Therefore, fragments 1–27 and 28–51 were coupled by NCL. The thioester of the N-terminal part was prepared by Boc chemistry on a Cys-SH resin, while the C-terminal peptide was synthesized using the conventional Fmoc/tBu protocol.

Correct pairing of cysteines and correct folding is essential for the biological activity of most proteins containing multiple disulfide bridges, among them AFP. This study used two strategies for cysteine side chains: uniform and semi-orthogonal protection of thiols. When the eight sulfhydryl groups were protected uniformly, air oxidation led to mainly forming three disulfide isomers. The major product was identical to AFPN, while the two minor products (misfolded isomers) were successfully refolded into the native state. In the case of semi-orthogonal protection, Trt in Fmoc or Meb in Boc chemistry was used for two pairs of cysteines and Acm for the other two pairs. All three possible isomers (globular, ribbon, and bead) were created during the first oxidation step. After isolation, the proteins were treated with iodine to remove Acm and oxidize sulfhydryl groups. Only two main products were generated in all three cases, so altogether, six different proteins were formed, and one of them proved to be identical to native AFP. It means semi-orthogonal protection of cysteine side chains decreased the theoretical number of disulfide isomers (105) to only 6.

The synthetic protein containing the native disulfide pattern (AFPnf) was identical to AFP from *A. giganteus* (AFPN). First, NMR investigation (^1^H, ^1^H–^15^N, and ^1^H–^13^C HSQC, and ^1^H–^1^H NOESY) showed the identity of the proteins. Second, contrary to misfolded variants (AFPmf1 and AFPmf2), AFPnf and AFPN equally inhibited the growth of *A. niger* in broth microdilution assay and reduced the *A. niger* colony diameter when cultivated on a solid medium. Third, enzymatic cleavage and MS analysis confirmed that the intermediate in semi-orthogonal protection strategy possessing globular disulfide connectivity was transformed into the native-patterned protein as expected.

Establishing AFP’s disulfide topology presented in this study is key to producing modified protein variants. The natural structure reservoir of antimicrobial peptides is highly versatile, and particularly among antifungal peptides, increased contents of cysteine residues can be observed.^37^ Thus, the synthetic methods discussed here can be applied to prepare AFP analogs to obtain more effective antifungal agents and study structure–activity relationships.

## Experimental Section

### AFP Production and Purification

AFPN was produced by *A. giganteus* ifGB0902 and purified according to Theis et al. with the following modifications.^38^ Five 1-L Erlenmeyer flasks each containing 200 mL of minimal medium (w/v: 0.3% NaNO_3_, 0.05% MgSO_4_ × 7H_2_O, 0.05% KCl, 0.005% FeSO_4_ × 7H_2_O, 2% d(+)-sucrose, 2.5% 1 M K_3_PO_4_-buffer (pH 5.8), 0.1% trace elements); trace elements (w/v: 0.9% ZnSO_4_ × 7H_2_O, 0.04% CuSO_4_ × 5H_2_O, 0.01% MnSO_4_ × H_2_O, 0.01% H_3_BO_3_, 0.01% Na_2_MoO_4_ × 2H_2_O) were inoculated with 1 × 10^8^ fresh conidia per flask. *A. giganteus* was first grown for 96 h at 28 °C under shaking conditions (200 rpm), followed by cultivation for a further 48 h at 37 °C and shaking at 200 rpm. The AFPN was purified from the cell-free supernatant as described for other cysteine-rich antifungal proteins from filamentous *Ascomycetes*.^21^ In brief, ultrafiltered (Ultracell 30 kDa, Millipore) supernatant was applied to a CM-Sepharose (Fast Flow, GE Healthcare Life Sciences) column, equilibrated in 10 mM Na_3_PO_4_, 25 mM NaCl, 0.15 mM EDTA (pH 6.6), and the protein was eluted with 0.5 M NaCl. The AFP-containing fractions were pooled, dialyzed (3.5 K MWCO, ThermoFisher Scientific) against ultrapure H_2_O, and sterilized (0.22 mm, Millex-GV, PVDF, Millipore). The protein concentration was determined spectrophotometrically, considering the respective molar extinction coefficient. The sample purity was checked by SDS-PAGE using silver staining. Isotopic ^15^N-labeling of AFPN for NMR analysis was achieved by replacing the nitrogen source in the minimal medium with 0.3% (w/v) Na^15^NO_3_ (Euriso-Top). The isotope-labeled AFPN (^15^N-AFPN) was produced and purified the same way as the unlabeled AFPN.

### Peptide Synthesis and Purification

Peptides were synthesized by the solid-phase method. Stepwise synthesis of AFP was attempted on a PL-Wang resin using a CEM Liberty Blue microwave-assisted peptide synthesizer and Fmoc/tBu chemistry. The coupling reagents included *N*,*N’*-diisopropyl carbodiimide and Oxyma. The C-terminal fragments for NCL were synthesized manually on preloaded Wang PS resin utilizing Fmoc/*t*Bu chemistry and dicyclohexyl carbodiimide/1-hydroxybenzotriazole coupling with a 3-fold excess of the reagents. The N-terminal fragment for NCL was prepared on the previously published Cys-SH resin utilizing Boc/Bzl chemistry.^17^ Briefly, Fmoc-Cys(Trt)-OH was coupled to 4-methylbenzhydrylamine resin, followed by the cleavage of Fmoc, and acetylation of the free amino group. Then the Trt group was cleaved by trifluoroacetic acid (TFA) from the cysteine thiol. The C-terminal amino acid was linked to the free SH group by applying double coupling in the presence of 4-dimethylaminopyridine as a catalyst. Peptides and proteins were detached from the Wang resins with a TFA/H_2_O/dithiothreitol (95%:5%:3% v/v:v/v:m/v) cleavage mixture. The N-terminal fragment was cleaved from the solid support with liquid HF in the presence of 2% (v/v) anisole and 8% (v/v) dimethyl sulfide scavengers at −5 to 0 °C. Crude peptides and proteins were purified, and analytical RP-HPLC checked the purity of the products. Samples were eluted using a linear gradient of organic (80% v/v MeCN/0.1% v/v TFA) solvent against aqueous (0.1% v/v TFA). The eluent was monitored at 220 nm. The identity of the products was verified by MS. NCL was performed in a 0.1 M NH_4_OAc buffer (pH 7.5) containing 3% (v/v) thiophenol at room temperature for 3–4 h. The precipitate was dissolved with the help of MeCN and guanidine hydrochloride, and RP-HPLC isolated the product. Analytical RP-HPLC checked the purity of the product, and MS verified its identity.

### Disulfide Bond Formation

The first method was the uniform protection of cysteine thiols. To oxidize sulfhydryl groups, the protein was dissolved in a 0.1 M NH_4_OAc buffer (pH 7.5) at 0.2 mg mL^–1^ concentration, and air oxygen was intensively stirred into the solution for 24 h. The mixture was then lyophilized three times to remove NH_4_OAc, and RP-HPLC isolated the product. As written above, the first two disulfide bonds formed in the semi-orthogonal protection strategy. The three AFP(Acm_2_)-C2 isomers were isolated from the mixture and treated with 5 equiv of iodine in 0.25 M HCl solution containing 60% (v/v) MeOH at 0.2 M protein concentration for 30 min. Excess iodine was reduced by ascorbic acid, and the mixture was subjected to RP-HPLC purification.

### NMR Spectroscopy

All NMR spectra were recorded using a Bruker Avance II spectrometer operated at 500.13 MHz ^1^H resonance frequency. Typical 90° RF flip angles were 9, 32, and 16 μs for ^1^H, ^15^N, and ^13^C channels. The samples (AFPnf and ^15^N-AFPN) were measured at 298 K using 20 mM acetate buffer adjusted to pH 4.5. Amounts of 1.5 mg of AFPnf and 2.8 mg of ^15^N-AFPN were dissolved in 275 μL of buffer containing 5% (v/v) D_2_O and filled into special Shigemi NMR tubes to obtain higher sensitivity. 1D ^1^H NMR spectra were recorded using “watergate” water suppression. ^1^H–^15^N HSQC spectra were recorded using the manufacturer’s “hsqcetf3gpsi2” pulse program using NS = 1024 (AFPnf) or two scans (^15^N-AFPN) per 128 or 256 increments, defining a 25 ppm ^15^N spectral window. The ^1^H–^13^C HSQC spectra were recorded using the “hsqcetgpsi2” pulse sequence using NS = 96 (AFPnf) or 128 scans (^15^N-AFPN) per 512 or 770 increments, defining a 65 ppm ^13^C spectral window. ^1^H–^1^H NOESY spectra were measured with 130 ms mixing times using the “noesygpph19” pulse sequence, equipped with ^15^N decoupling during acquisition for the ^15^N-AFPN sample. For AFPnf 64 scans and 512 increments, while for ^15^N-AFPN 128 scans and 640 increments were applied.

### Identification of Disulfide Bond Pattern by MS

The digested samples were analyzed on a Thermo Q-exactive plus UPLC system coupled with a Micromass Q-TOF premier mass spectrometer. The mass spectrometer was operated in automated DDA mode. All acquired data were processed, and peak lists were generated with MSconvert software (http://www.proteowizard.org/download.html). The resulting.mgf file was processed and sorted according to mass intensities using Microsoft Excel 2010 software. The first 50 most intense peaks were analyzed with disulfide bridge analyzer software (http://prospector.ucsf.edu/prospector/cgi-bin/msform.cgi?form=msbridgestandard).

### Antifungal Activity Assays

All antifungal activity assays were performed in yeast extract–peptone–glucose medium (YPG, w/v: 1% peptone, 0.3% yeast extract, 2% glucose). The antifungal activities of AFPN and synthetic variants having native (AFPnf) or altered (AFPmf1 and AFPmf2) disulfide bond patterns were compared in a microtiter plate bioassay against *A. niger* SZMC 601 (Szeged Microbial Collection, University of Szeged, Szeged, Hungary) according to Sonderegger et al. with the following modifications.^21^ A 100 μL protein solution (1.56–100 μg mL^–1^ in 2-fold dilution in YPG) was mixed with 100 μL of conidium suspension (2 × 10^4^ conidia mL^–1^ in YPG), resulting in a final protein concentration ranging from 0.78 to 50 μg mL^–1^ in the wells of a flat-bottom microtiter plate (VWR Tissue Culture Plates, 96 wells-F). After incubation for 48 h at 25 °C without shaking, the fungal growth was determined by measuring the optical density at 620 nm with a microtiter plate reader (SPECTROstar Nano, BMG Labtech) in well-scanning mode. For the calculation of % growth of the treated samples, the absorbance of the untreated control (100 μL YPG mixed with 100 μL of 2 × 10^4^ conidia mL^–1^ in YPG was referred to as 100% of growth. The MIC was defined as the lowest antifungal protein concentration that led to ≤5% fungal growth. To determine the growth inhibition of *A. niger* grown on a solid medium, 2 × 10^3^ conidia (in 5 μL YPG) were spotted on YPG agar (2% (w/v) agar) containing 1 μg mL^–1^ of protein. Colony growth was inspected visually after 48 h of incubation at 25 °C. All antifungal susceptibility tests were repeated three times. An unpaired *t* test was performed for the statistical analysis using GraphPad QuickCalcs.

## Supporting Information Available

Chemicals; details of synthetic procedures; RP-HPLC analysis of intermediates and products; MS analysis of the synthesized compounds; ^1^H NMR and ^1^H–^13^C HSQC spectra of native and synthetic AFPs; MS/MS fragmentation spectra of pairs of peptide fragments to identify disulfide bond pattern (PDF)
