# Supplementary material for: Confirmation of the Disulfide Connectivity and Strategies for Chemical Synthesis of the Four-Disulfide-Bond-Stabilized Aspergillus giganteus Antifungal Protein, AFP
Source: J Nat Prod. 2023 Feb 27;86(4):782–90. doi: 10.1021/acs.jnatprod.2c00954 (PMC10152477; doi:10.1021/acs.jnatprod.2c00954)
Supplement: Supplementary file 1 — np2c00954_si_001.pdf [file np2c00954_si_001.pdf]

Supporting Information

Confirmation of the Disulfide Connectivity and Strategies for Chemical Synthesis of the Four-Disulfide Bond Stabilized *Aspergillus giganteus* Antifungal Protein, AFP

Györgyi Váradi,[1]* Gyula Batta,[2] László Galgóczy,[3,4,5] Dorottya Hajdu,[2] Ádám Fizil,[2] András Czajlik,[2,6] Máté Virágh,[4] Zoltán Kele,[1] Vera Meyer,[7] Sascha Jung,[7] Florentine Marx,[5] Gábor K. Tóth[1,8]

[1]Department of Medical Chemistry, University of Szeged, Szeged 6720, Hungary

[2]Department of Organic Chemistry, University of Debrecen, Debrecen 4010, Hungary

[3]Department of Biotechnology, University of Szeged, Szeged 6726, Hungary

[4]Institute of Biochemistry, Biological Research Centre, Eötvös Loránd Research Network, Szeged 6726, Hungary

[5]Institute of Molecular Biology, Biocenter, Medical University of Innsbruck, Innsbruck 6020, Austria

[6]Faculty of Information Technology and Bionics, Pázmány Péter Catholic University, Budapest 1083, Hungary

[7]Department of Applied and Molecular Microbiology Technische Universität Berlin, Institute of Biotechnology, Berlin 13355, Germany

[8]MTA-SZTE Biomimetic Systems Research Group, University of Szeged, Szeged 6720, Hungary

Table of contents

1. **Chemical synthesis** S3

2. **RP-HPLC analysis and purification** S5

3. **MS analysis of the synthesized compounds** S15

4. **NMR investigation of AFPs** S21

5. **Identification of disulfide bond pattern by MS** S23

6. **Reference** S26

1. Chemical synthesis

Chemicals

All amino acid derivatives were purchased from Chem-Impex (Wood Dale, IL, USA), Iris Biotech GMBH (Marktredwitz, Germany), or Bachem AG (Bubendorf, Switzerland). PL-Wang resin was obtained from Varian, Inc., 4-methylbenzhydrylamine (MBHA) resin from Iris Biotech GMBH (Marktredwitz, Germany), and Wang PS resin from Senn Chemicals AG (Dielsdorf, Switzerland). DCC was purchased from Iris Biotech GMBH (Marktredwitz, Germany), 4-dimethylaminopyridine (4-DMAP) from Fluka (Sigma-Aldrich, Budapest, Hungary), and anhydrous 1-hydroxybenzotriazole (HOBt) from Abcr GmbH (Karlsruhe, Germany). Solvents and reagents for microwave-assisted peptide synthesis were purchased from the following suppliers: N-methylpyrrolidone (NMP) from Iris Biotech GMBH (Marktredwitz, Germany), dimethylformamide (DMF) from Merck KGaA (Darmstadt, Germany), diisopropylcarbodiimide (DIC) and Oxyma from Fluorochem Ltd (Hadfield Derbyshire, UK). Solvents and reagents for manual solid-phase synthesis were obtained from the following companies: dichloromethane (DCM), dimethylformamide (DMF), methanol, and piperazine from Alfa Aesar (Thermo Fisher Scientific GmbH, Kandel, Germany), and trifluoroacetic acid (TFA) and dithiothreitol (DTT) from Fluorochem Ltd (Hadfield Derbyshire, UK). HPLC grade TFA and acetonitrile (ACN) were obtained from Sigma-Aldrich (St. Louis, MO, USA). All other chemicals used were of the highest grade available.

Stepwise synthesis of AFP

Microwave-assisted solid-phase peptide synthesis was applied for the ***stepwise synthesis*** of AFP using an automated Liberty Blue^{TM} peptide synthesizer (CEM Corporation, Matthews, NC, USA) and Fmoc chemistry. PL-Wang resin (Varian, Palo Alto, CA, USA) having a substitution level of 0.6 mmol g^{-1} was used as solid support. The C-terminal amino acid (Fmoc-Cys(Trt)-OH) was attached to the resin manually applying DCC/HOBt double coupling in the presence of a 4-fold excess of the amino acid and the reagents. A 0.2 equiv. of 4-dimethylaminopyridine (4-DMAP) as a catalyst was also added to the reaction mixture. The formed preloaded resin was used for the synthesis of the protein. The side-chain protecting groups were the following: Asn(Trt), Asp(OBu^t), Cys(Trt), Glu(OBu^t), Lys(Boc), Ser(Bu^t), Thr(Bu^t), and Tyr(Bu^t). A standard

DIC/Oxyma protocol was applied. The protein was cleaved from the polymer with the use of a TFA/water/dithiothreitol (DTT) (95% v/v/5% v/v/3% m/v) mixture for 3 hours, and it was precipitated with ice-cold diethyl ether. The precipitated peptide was collected by filtration and dried.

Synthesis of AFP by NCL

The ***N-terminal fragment for native chemical ligation*** was synthesized on the previously published Cys-SH resin.[1] Briefly, Fmoc-Cys(Trt)-OH was attached to MBHA resin followed by cleavage of the Fmoc group and acetylation of the free amino group. Then the trityl group was cleaved to form the Cys-SH resin. DCC/HOBt double coupling in the presence of a 4-fold excess of the amino acid and the reagents, and a 0.2 equiv. of 4-DMAP were used to couple the C-terminal lysine to the resin. The rest of the N-terminal fragment was synthesized according to standard DCC/HOBt protocol. The peptide was cleaved from the resin with the use of 8% (v/v) DMS, and 2% (v/v) anisole in liquid HF at -5-0 °C for 45 min. After the treatment with HF, the peptide was dissolved from the resin using 10% (v/v) acetic acid and lyophilized.

The ***C-terminal fragment for NCL*** was synthesized in solid phase using Fmoc chemistry and preloaded Wang PS resin (Senn Chemicals AG, Switzerland) having a substitution level of 1.2 mmol g^{-1} following standard DCC/HOBt protocol. The product was detached from the resin with a TFA/water/dithiothreitol (DTT) (95% v/v/5% v/v/3% m/v) cocktail at room temperature for 3 h. The resin was removed and the peptide was precipitated by the addition of ice-cold diethyl ether. The precipitated peptide was collected by filtration and dried.

NCL of the peptide fragments was carried out in a 0.1 M ammonium acetate buffer (pH 7.5) containing 3% (v/v) thiophenol at room temperature for 3-4 hours. In a typical experiment, 5 mg of the N-terminal fragment was dissolved in 2 mL of the buffer, followed by the addition of 60 μL of thiophenol. An equimolar amount of the C-terminal fragment was added to it, and the mixture was stirred for 3-4 hours at room temperature. Ligation was followed by analytical RP-HPLC. After completion of the reaction, the white precipitate was dissolved with the help of ACN and guanidine hydrochloride, and the full-length protein was isolated by semi-preparative RP-HPLC.

2. RP-HPLC analysis and purification

Crude peptides and ligation products were analyzed and purified by semi-preparative RP-HPLC using a solvent system of (A) 0.1% (v/v) TFA and (B) 80% (v/v) ACN, 0.1% (v/v) TFA. Purification was performed either on a Phenomenex Jupiter C18 10μ column (15 × 250 mm) at a flow rate of 4 mL min^{-1} or on a Vydac Protein & Peptide C18 column (10 × 250 mm) at a flow rate of 3 mL min^{-1}. The HPLC apparatus was made by Knauer (Berlin, Germany). Absorbance was detected at 220 nm in all cases. The purity of the peptides was evaluated by analytical RP-HPLC either on a 4.6 × 250 mm Phenomenex Luna 10μ C18 100A column or on a Vydac Protein & Peptide C18 column at a flow rate of 1 mL min^{-1} using an HP 1100 HPLC system made by Hewlett Packard (Palo Alto, CA, USA).

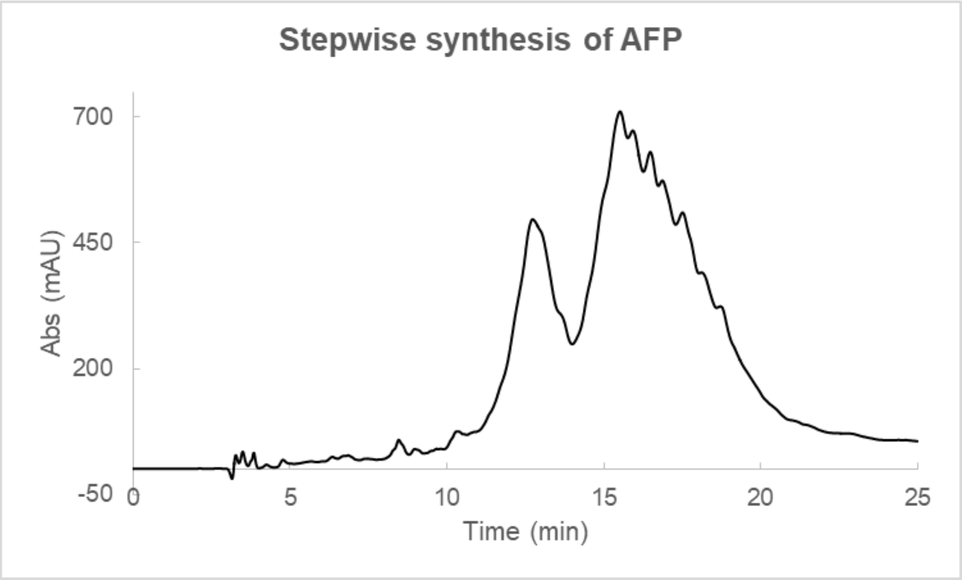

Figure S1. RP-HPLC profile of the product of stepwise AFP synthesis. (Side chains of cysteines were protected uniformly.) Gradient: 5-80% (B) in 25 min.

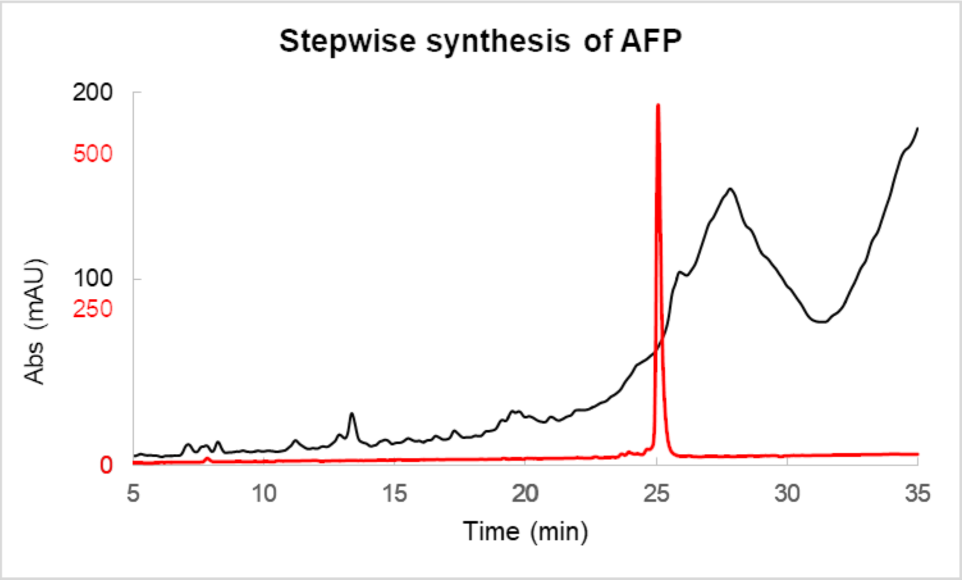

Figure S2. RP-HPLC profile of the product of stepwise AFP synthesis. (Side chains of cysteines were protected uniformly.) The chromatogram of native AFP for comparison is shown in red. Gradient: 5-40% (B) in 35 min.

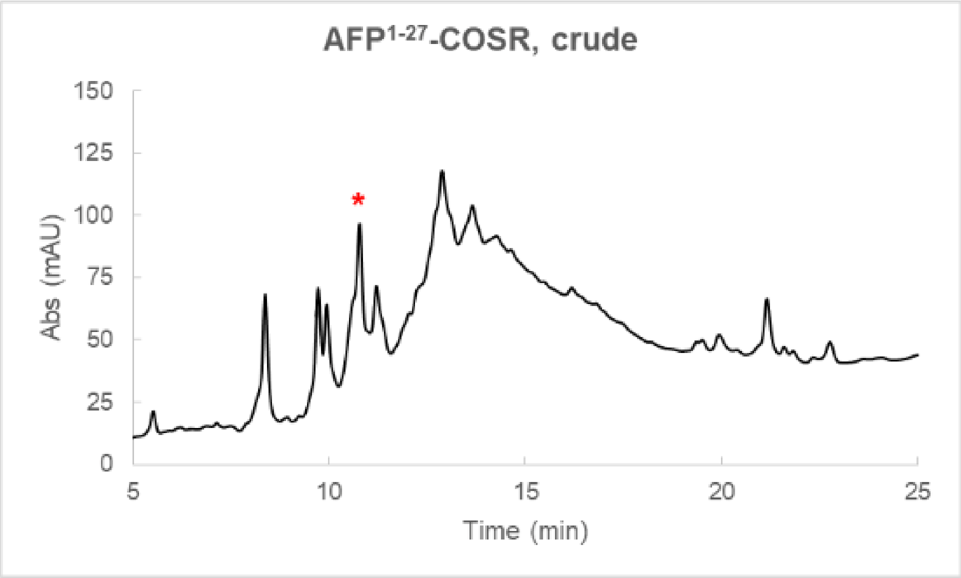

Figure S3. RP-HPLC profile of AFP's N-terminal thioester peptide fragment before purification. The desired peptide is labeled with a red asterisk. (Side chains of cysteines were protected uniformly.) Gradient: 5-80% (B) in 25 min.

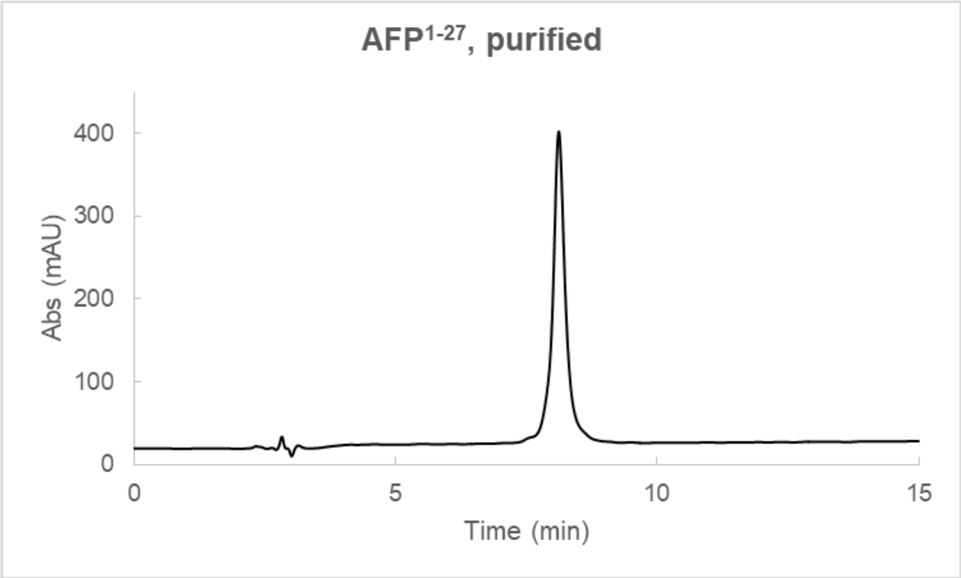

Figure S4. RP-HPLC profile of AFP's N-terminal thioester peptide fragment after purification. (Side chains of cysteines were protected uniformly.) Gradient: 18-33% (B) in 15 min.

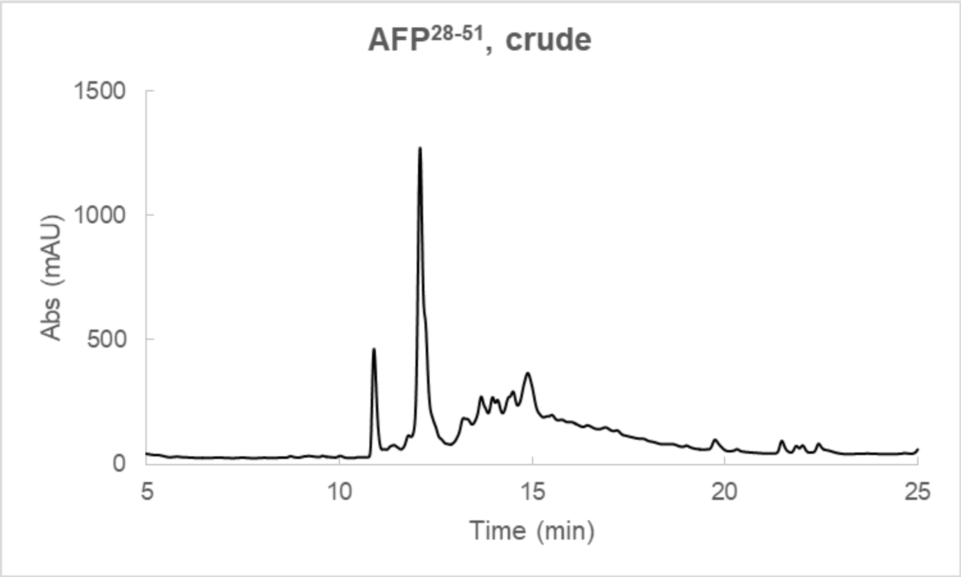

Figure S5. RP-HPLC profile of AFP's C-terminal peptide fragment before purification. (Side chains of cysteines were protected uniformly.) Gradient: 5-80% (B) in 25 min.

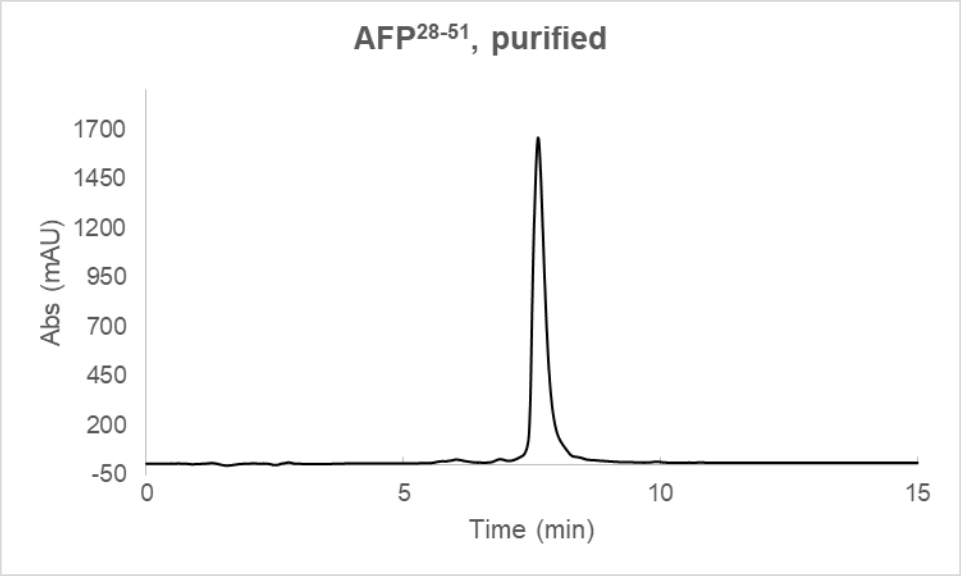

Figure S6. RP-HPLC profile of AFP's C-terminal peptide fragment after purification. (Side chains of cysteines were protected uniformly.) Gradient: 22-37% (B) in 15 min.

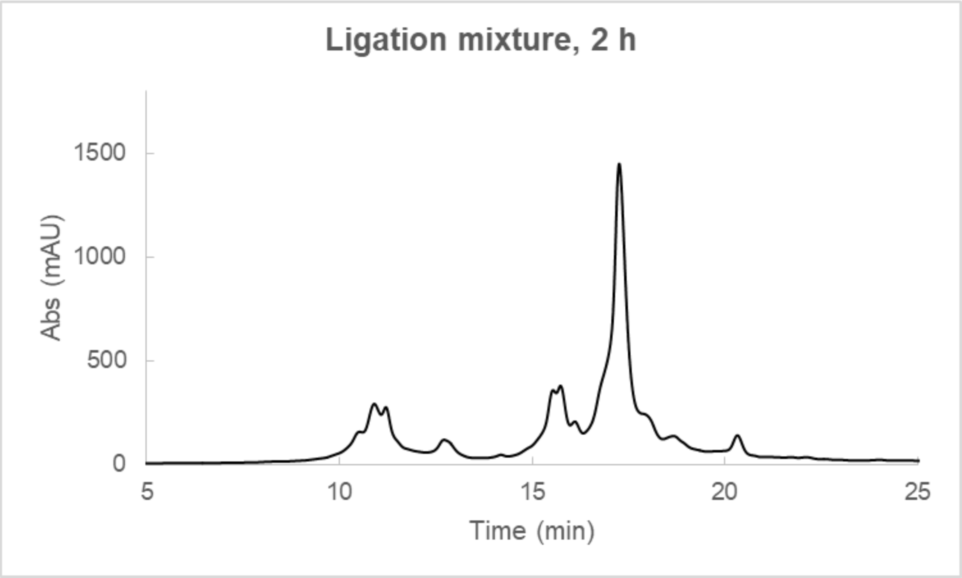

Figure S7. RP-HPLC profile of the ligation mixture of AFP[1-27] and AFP[28-51] in the synthesis of AFP in 2 hours. Gradient: 15-40% (B) in 25 min.

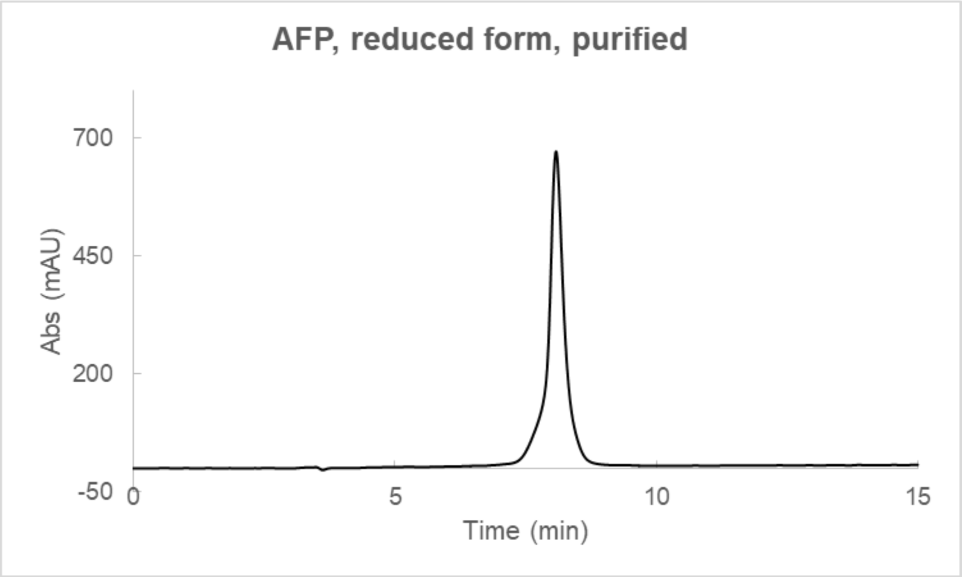

Figure S8. RP-HPLC profile of purified AFP in reduced form. Gradient: 24-39% (B) in 15 min.

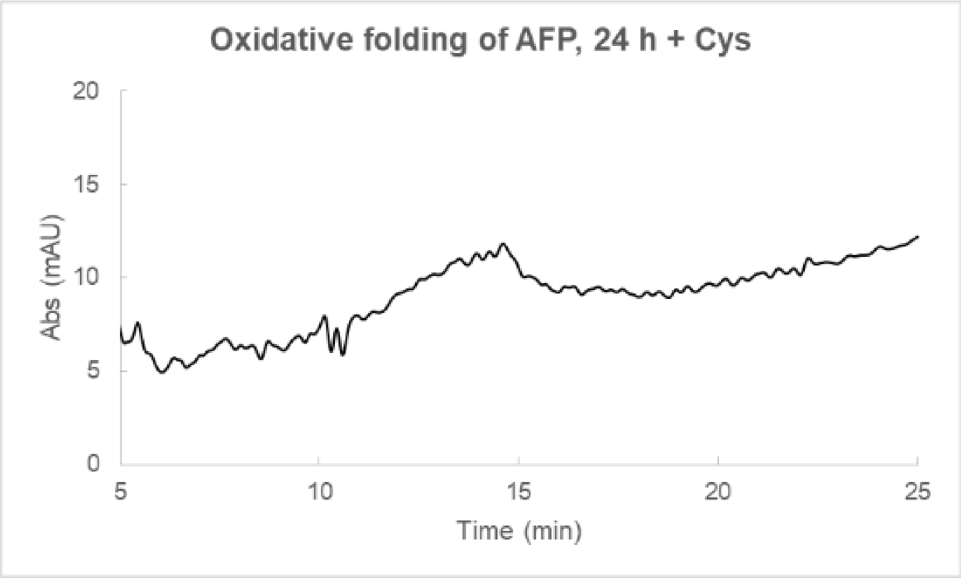

Figure S9. RP-HPLC profile of AFP's oxidative folding reaction mixture in the presence of cysteine in 24 h. Gradient: 15-40% (B) in 25 min.

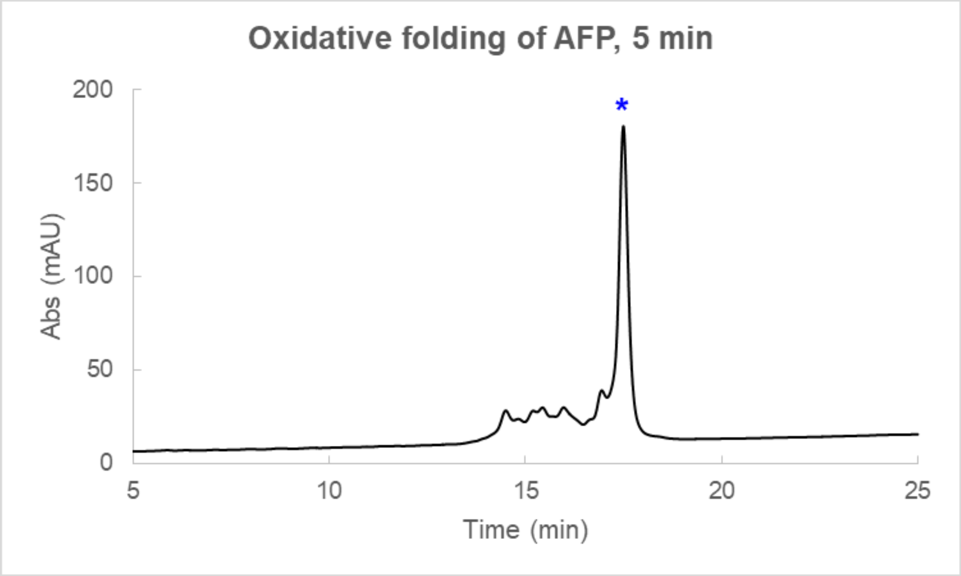

Figure S10. RP-HPLC profile of AFP's oxidative folding in 5 min. The reduced form of AFP is labeled with a blue asterisk. Gradient: 15-40% (B) in 25 min.

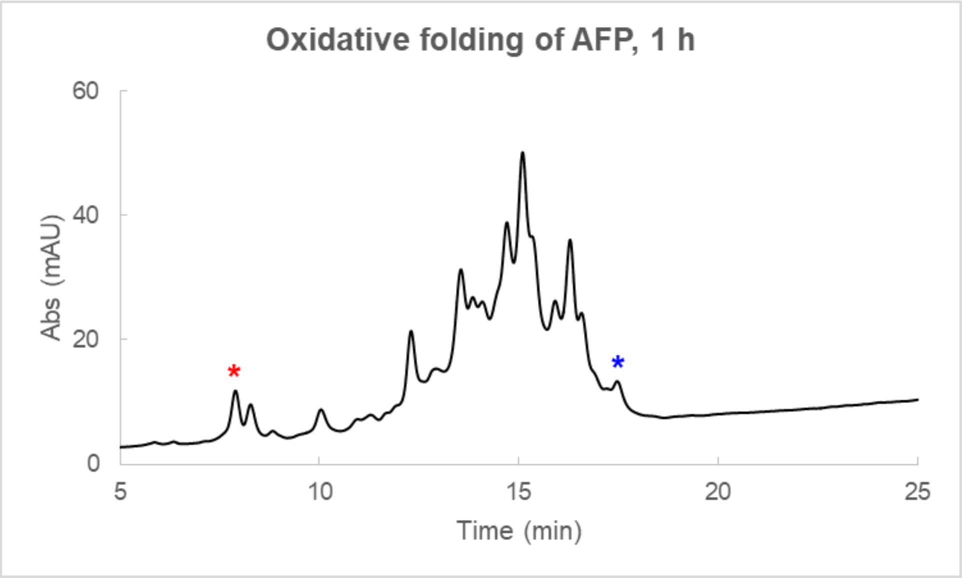

Figure S11. RP-HPLC profile of AFP's oxidative folding in 1 h. The reduced (blue) and the oxidized (red) forms of AFP are labeled with asterisks. Gradient: 15-40% (B) in 25 min.

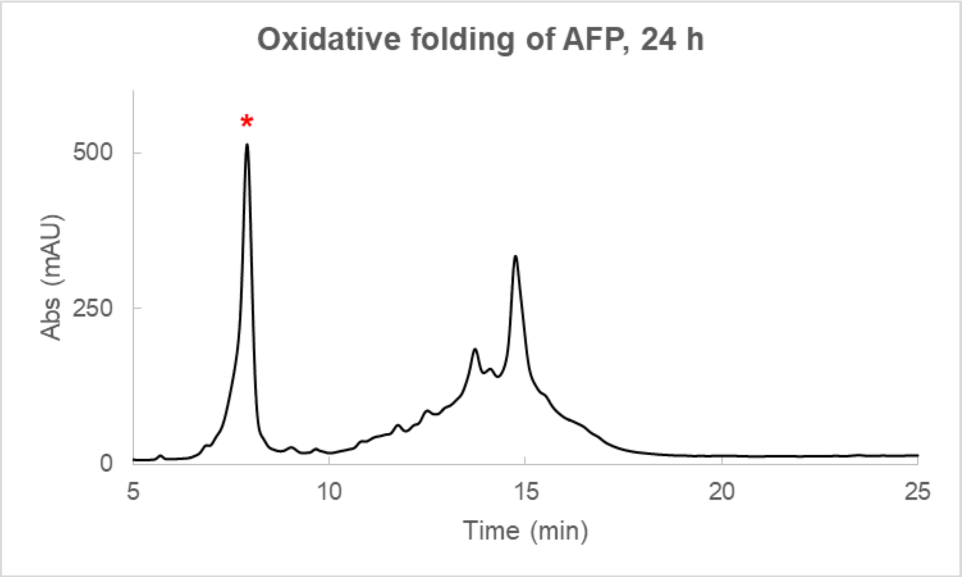

Figure S12. RP-HPLC profile of AFP's oxidative folding in 24 h. The oxidized form of AFP is labeled with a red asterisk. Gradient: 15-40% (B) in 25 min.

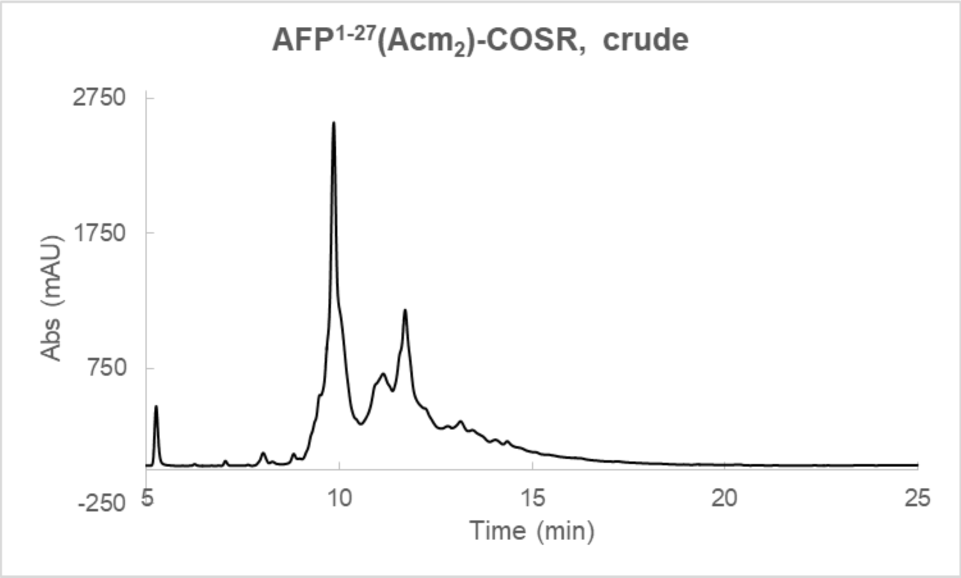

Figure S13. RP-HPLC profile of AFP's N-terminal thioester peptide fragment before purification. (Side chains of cysteines were protected semi-orthogonally.) Gradient: 5-80% (B) in 25 min.

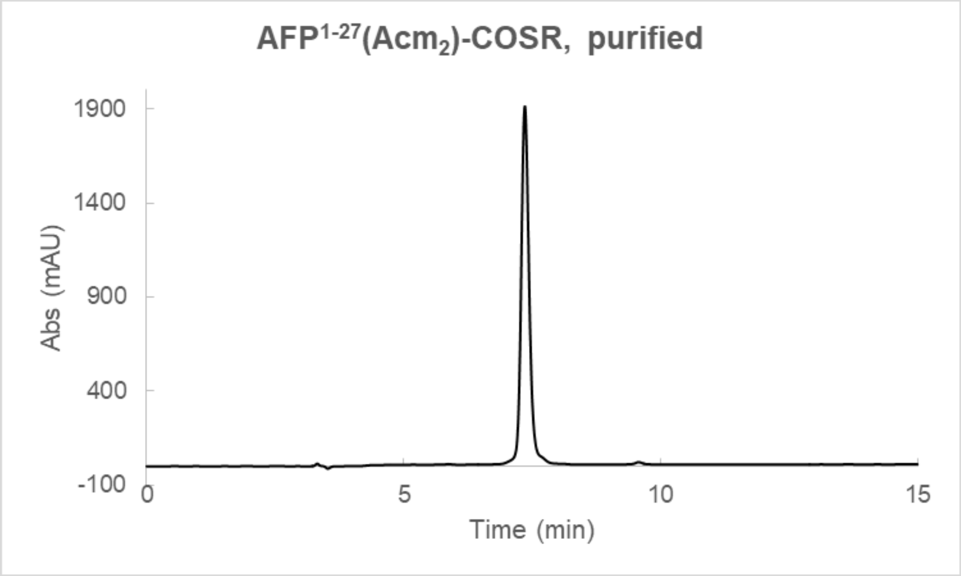

Figure S14. RP-HPLC profile of AFP's N-terminal thioester peptide fragment after purification. (Side chains of cysteines were protected semi-orthogonally.) Gradient: 17-32% (B) in 15 min.

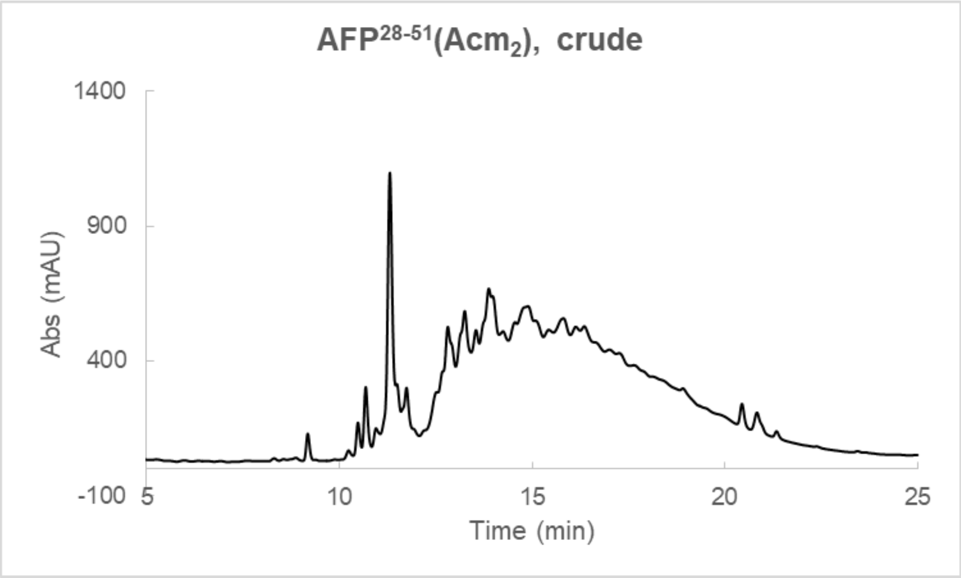

Figure S15. RP-HPLC profile of AFP's C-terminal peptide fragment before purification. (Side chains of cysteines were protected semi-orthogonally.) Gradient: 5-80% (B) in 25 min.

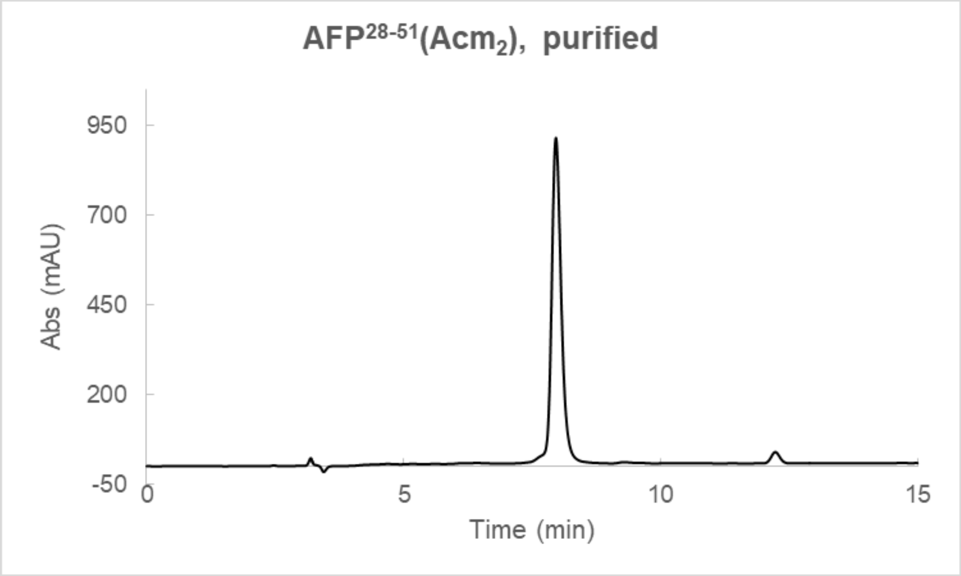

Figure S16. RP-HPLC profile of AFP's C-terminal peptide fragment after purification. (Side chains of cysteines were protected semi-orthogonally.) Gradient: 21-36% (B) in 15 min.

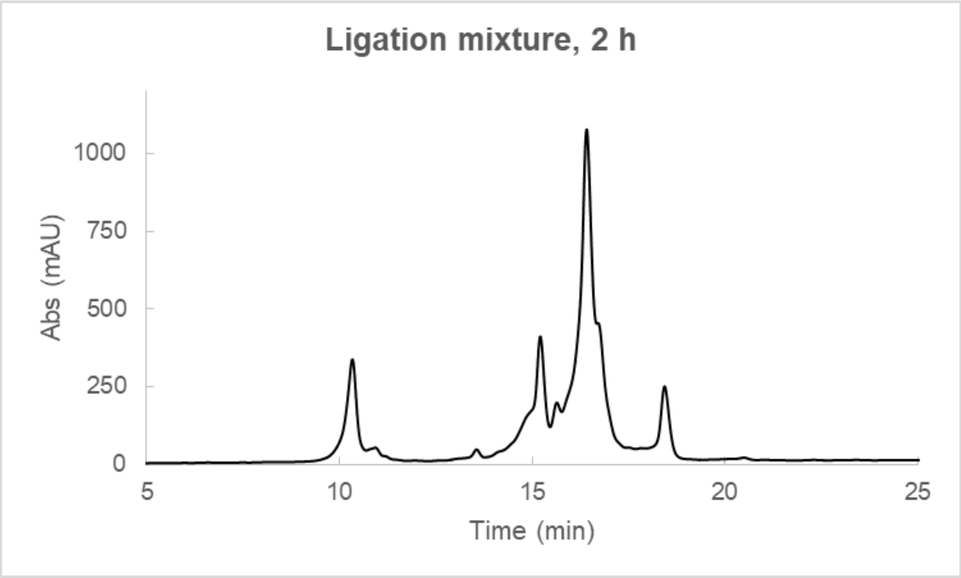

Figure S17. RP-HPLC profile of the ligation mixture in the synthesis of AFP(Acm$_4$) in 2 hours. Gradient: 15-40% (B) in 25 min.

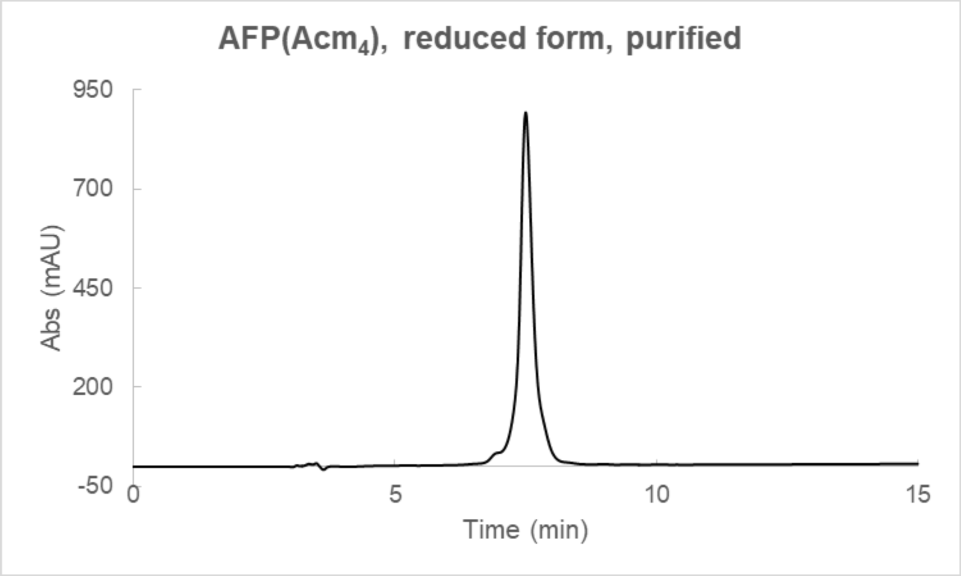

Figure S18. RP-HPLC profile of purified AFP(Acm$_4$) in reduced form. Gradient: 24-39% (B) in 15 min.

3. MS analysis of the synthesized compounds

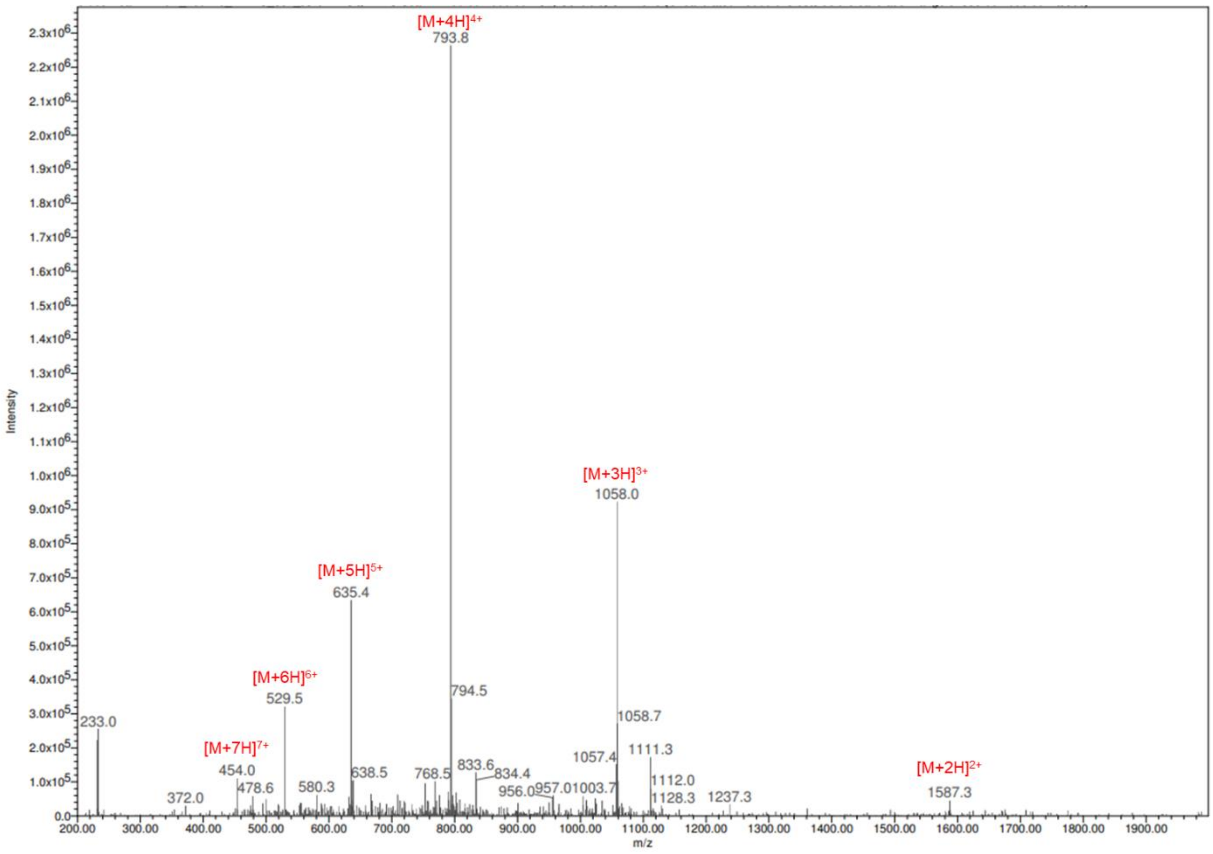

Figure S19. Mass spectrum of AFP$^{1\text{-}27}$-COSR, AFP's N-terminal thioester peptide fragment. (Side chains of cysteines were protected uniformly.)

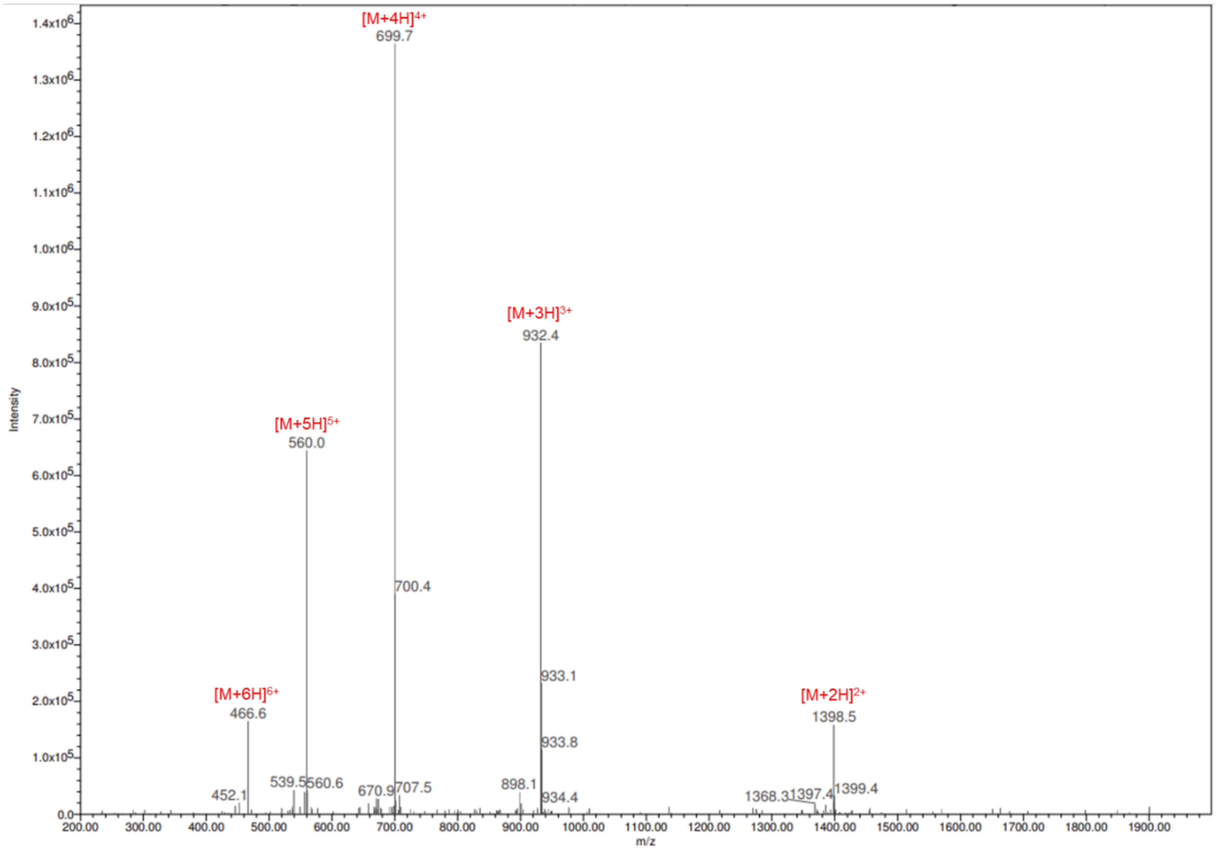

Figure S20. Mass spectrum of AFP$^{28\text{-}51}$, AFP's C-terminal peptide fragment. (Side chains of cysteines were protected uniformly.)

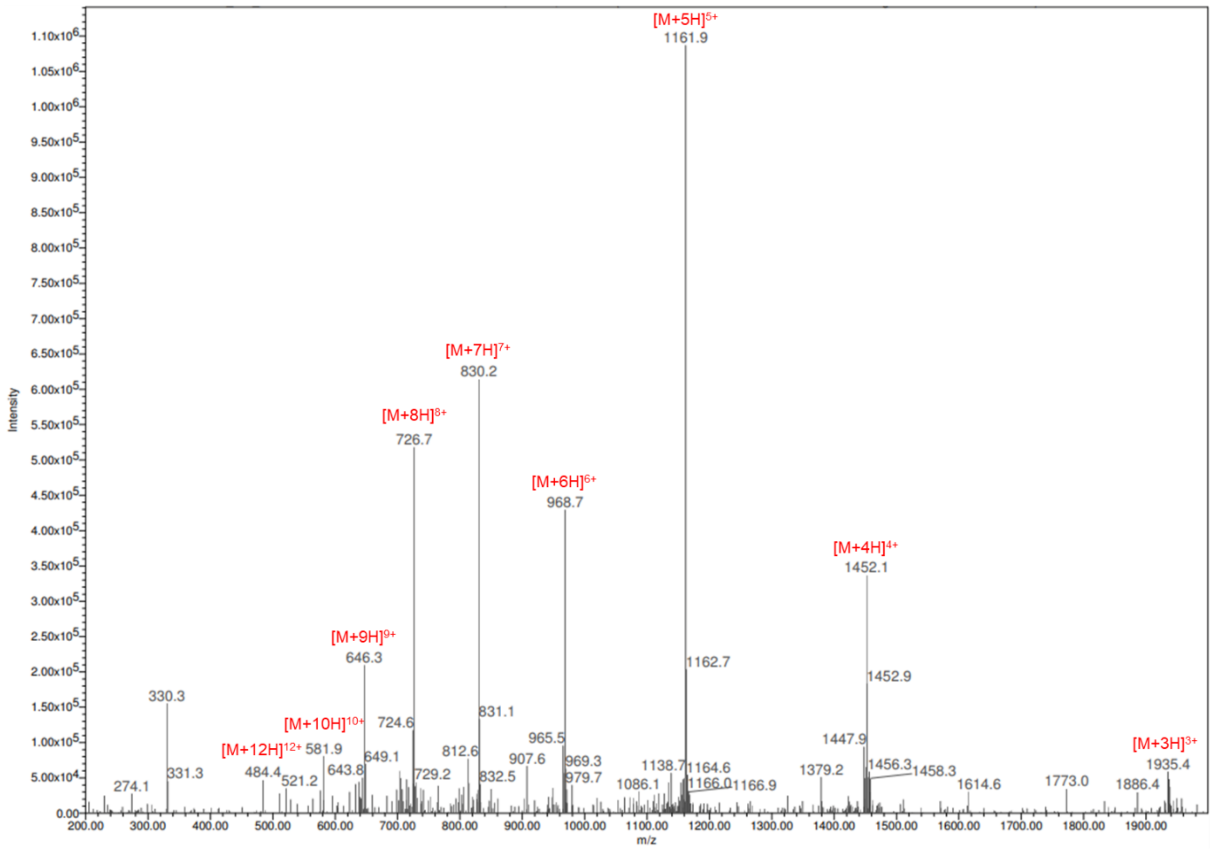

Figure S21. Mass spectrum of the reduced form of AFP. (Side chains of cysteines were protected uniformly.)

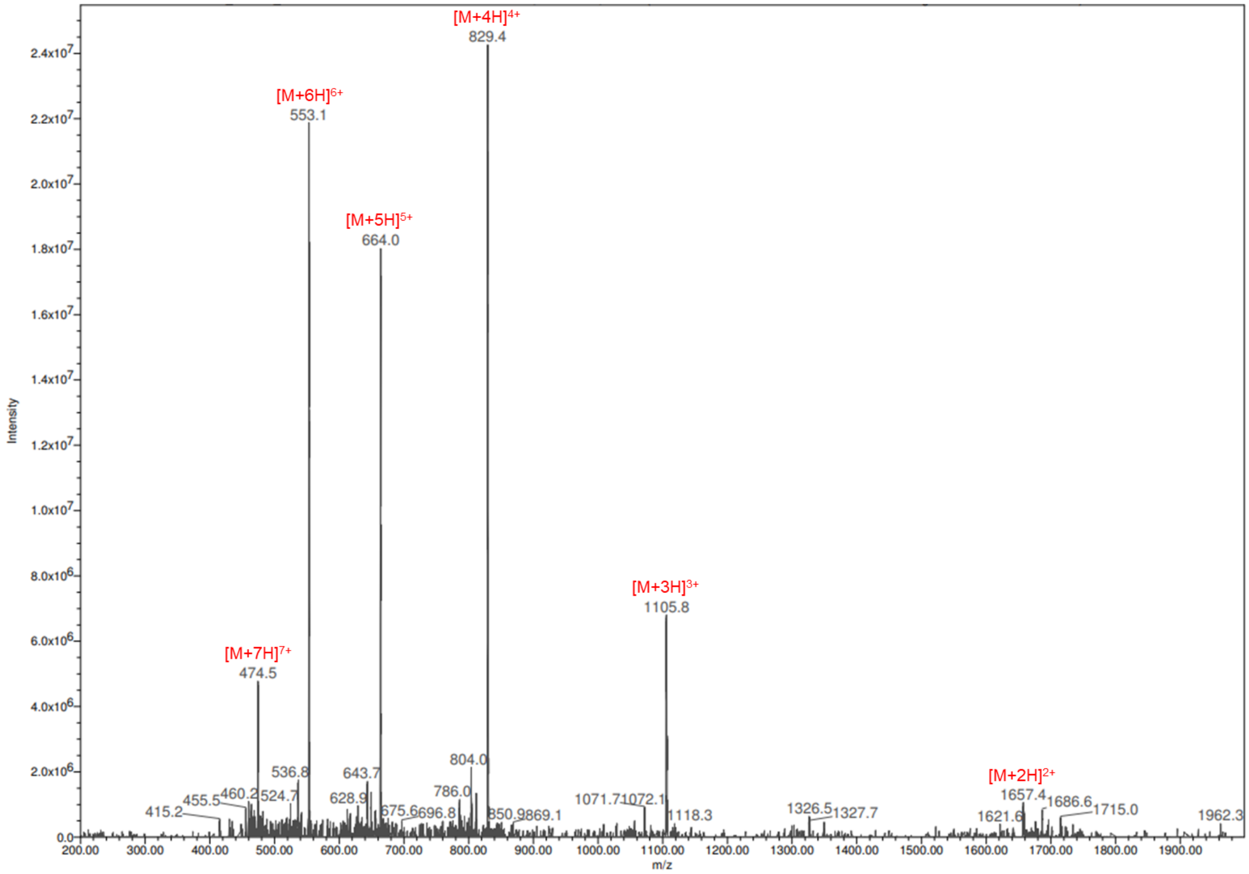

Figure S22. Mass spectrum of AFP$^{1\text{-}27}$(Acm$_2$)-COSR, AFP's N-terminal thioester peptide fragment. (Side chains of cysteines were protected semi-orthogonally.)

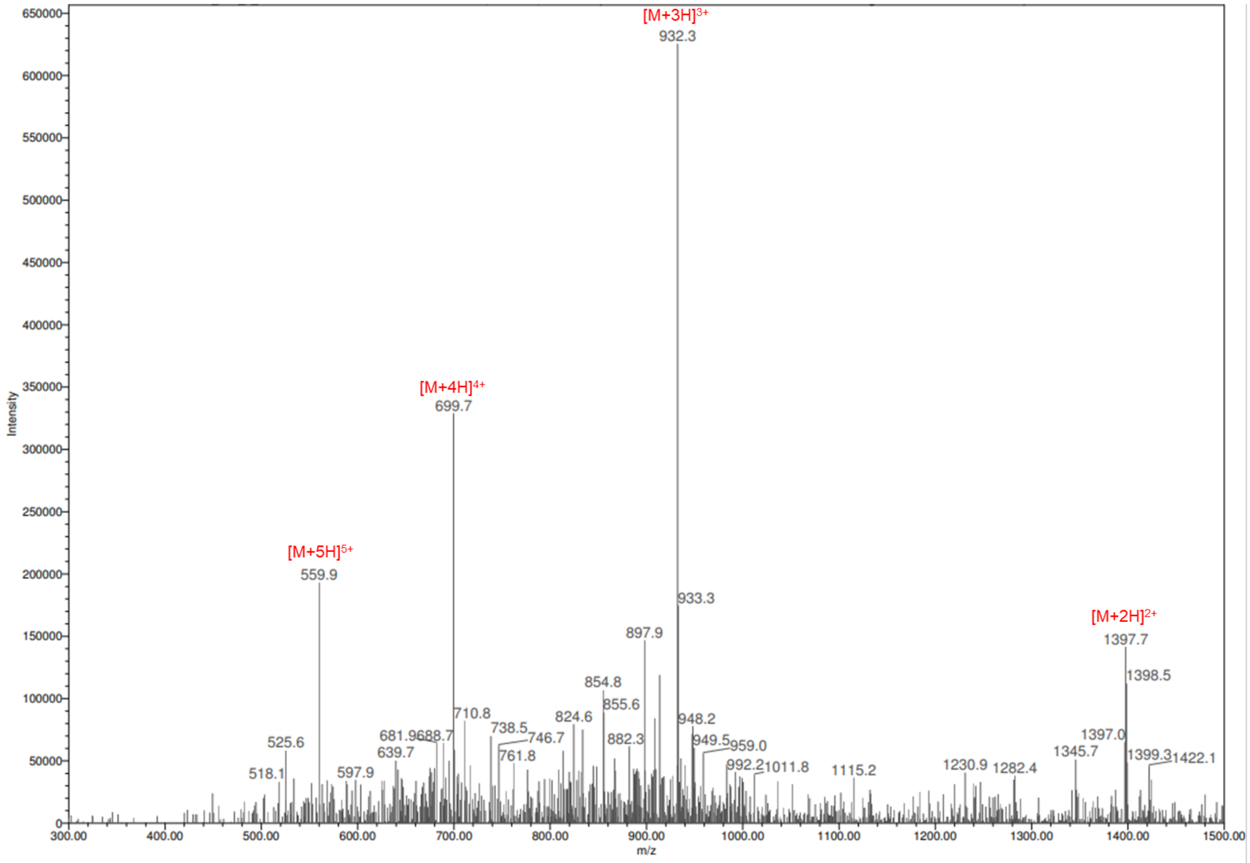

Figure S23. Mass spectrum of AFP$^{28\text{-}51}$(Acm$_2$), AFP's C-terminal peptide fragment. (Side chains of cysteines were protected semi-orthogonally.)

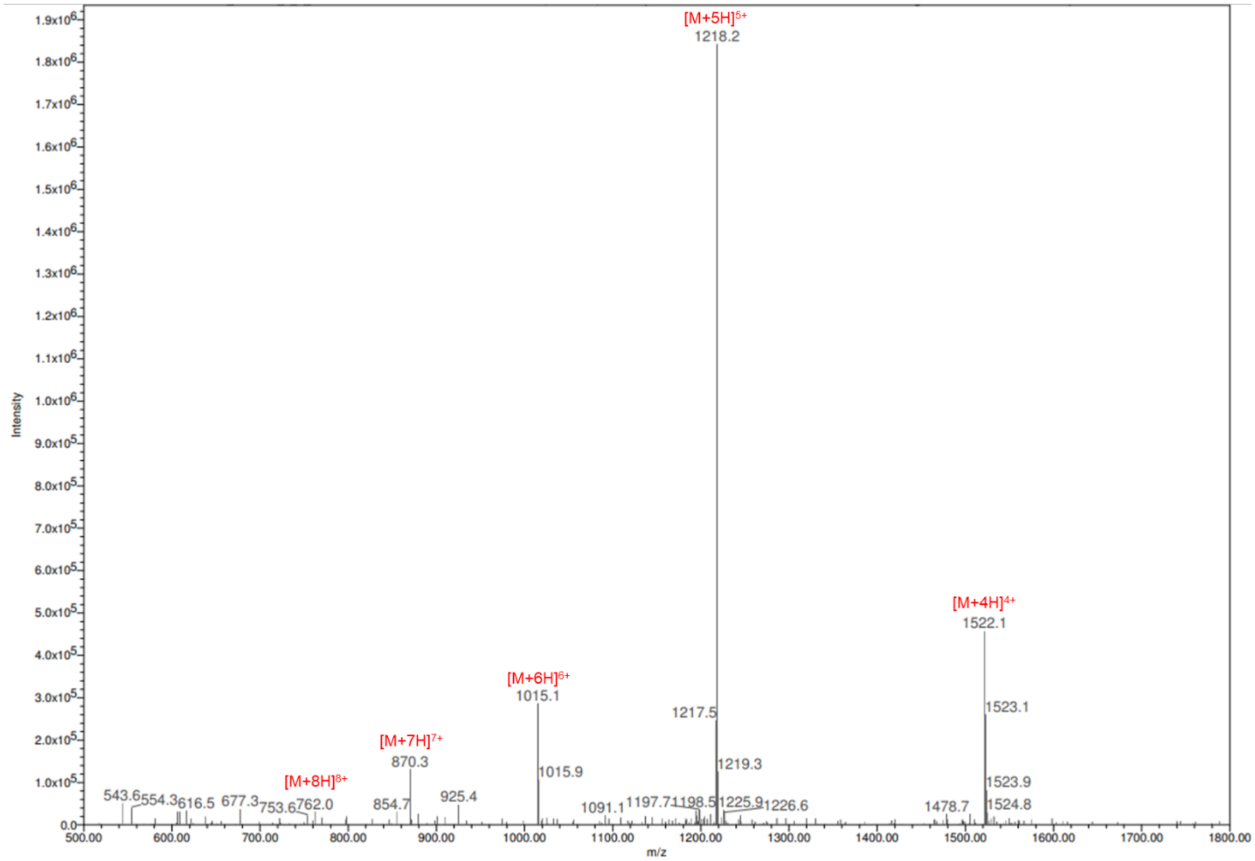

Figure S24. Mass spectrum of the reduced form of AFP(Acm_4). (Side chains of cysteines were protected semi-orthogonally.)

4. NMR investigation of AFPs

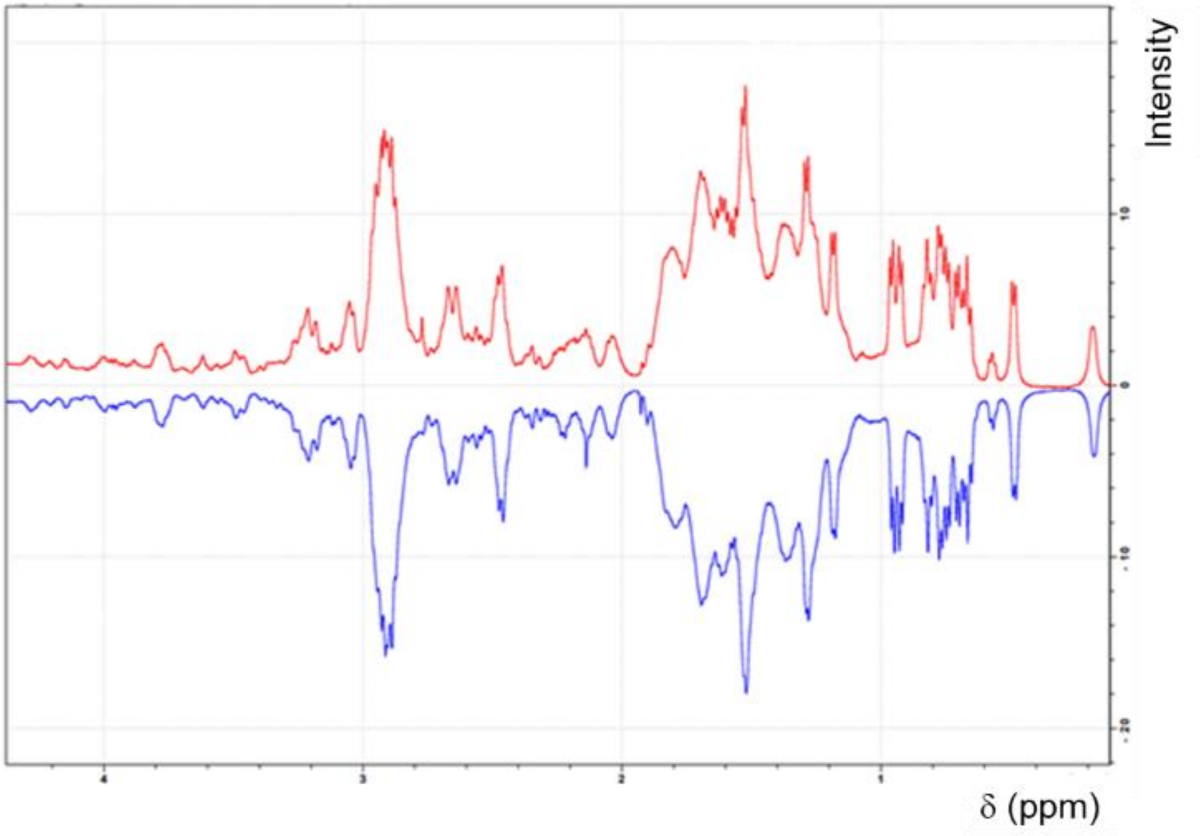

Figure S25. ^{1}H-NMR spectra of the native (AFPN) (red) and synthetic (AFPnf) (blue) AFP.

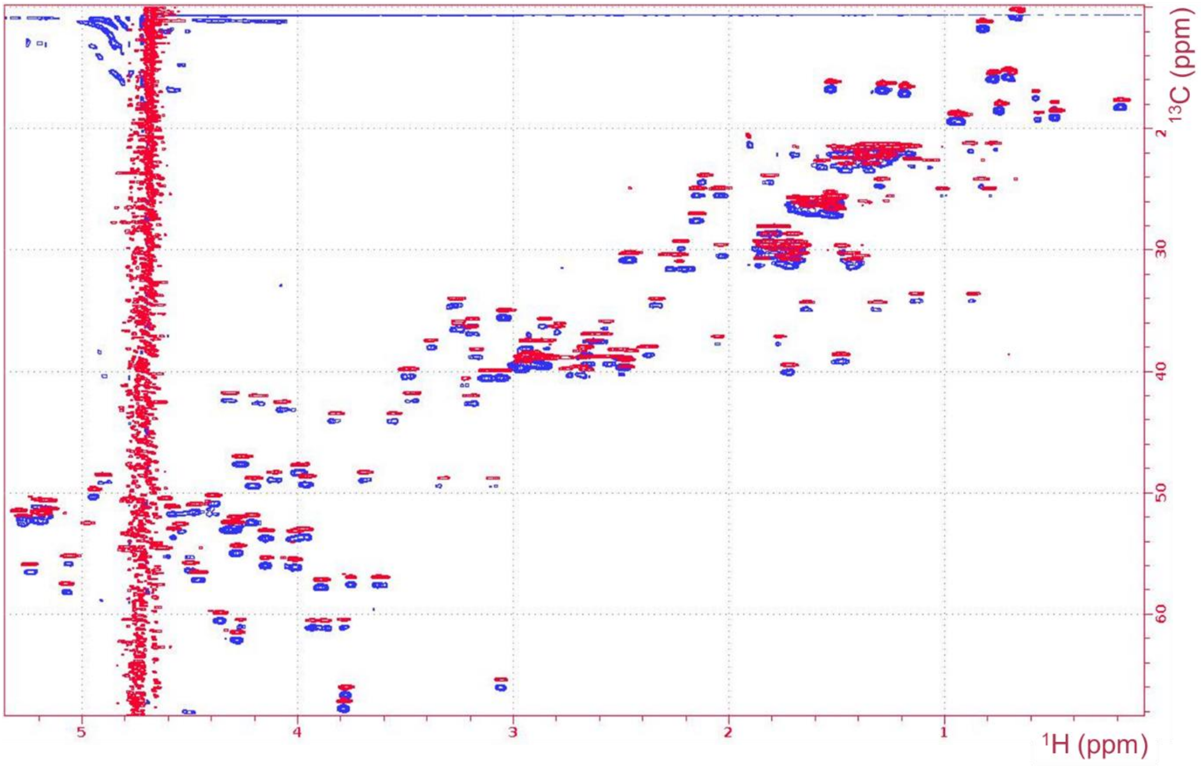

Figure S26. Comparison of the partial ^{1}H-^{13}C HSQC spectra of AFPs: ^{15}N-labelled AFP (AFPN) (red) and synthetic AFP (AFPnf) (blue). The two spectra are slightly shifted for better visibility.

5. Identification of disulfide bond pattern by MS

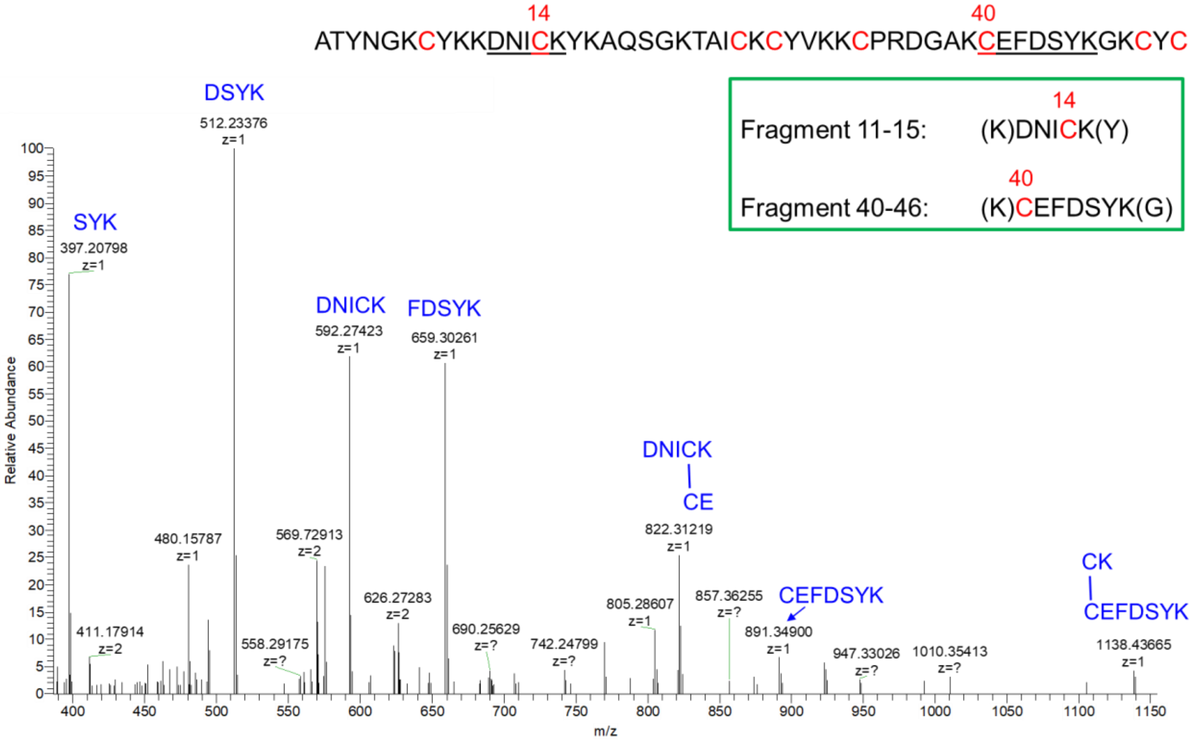

Figure S27. MS/MS fragmentation spectrum of the 494.2079^{3+} *m/z* ion corresponding to the DNICK-CEFDSYK pair of peptide fragments at a collision energy of 27 eV.

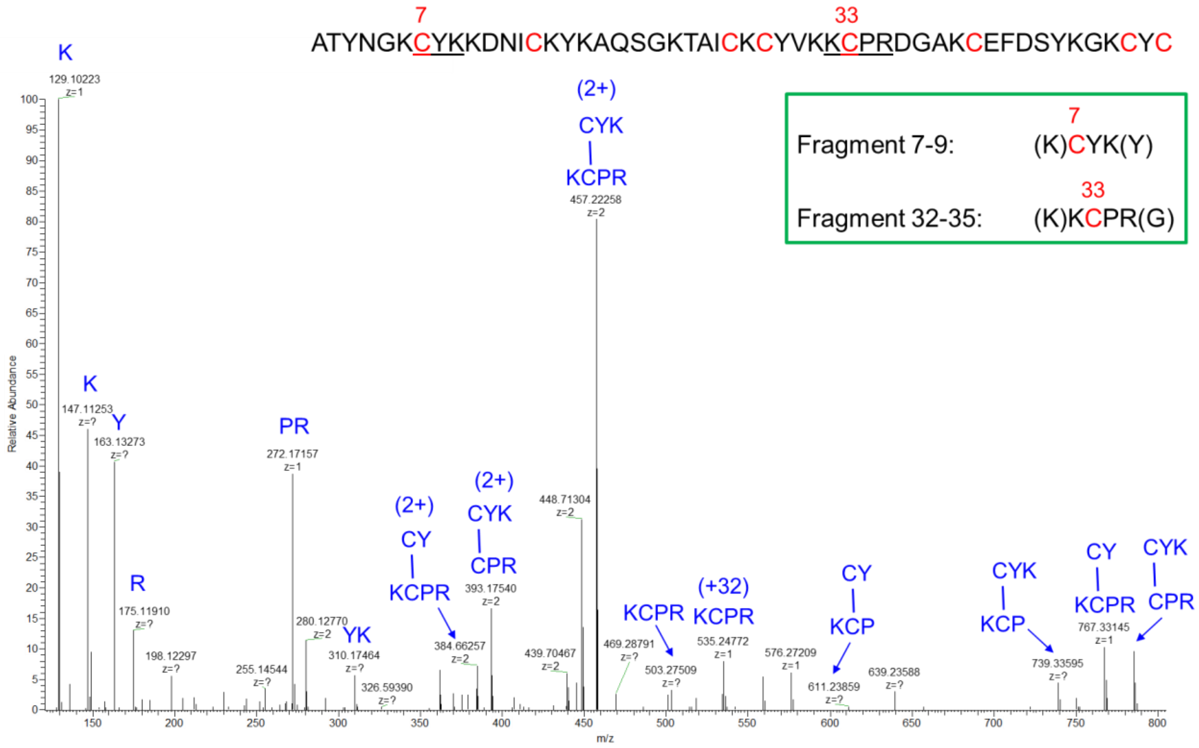

Figure S28. MS/MS fragmentation spectrum of the 457.2225^{2+} *m/z* ion corresponding to the CYK-KCPR pair of peptide fragments at a collision energy of 27 eV.

A

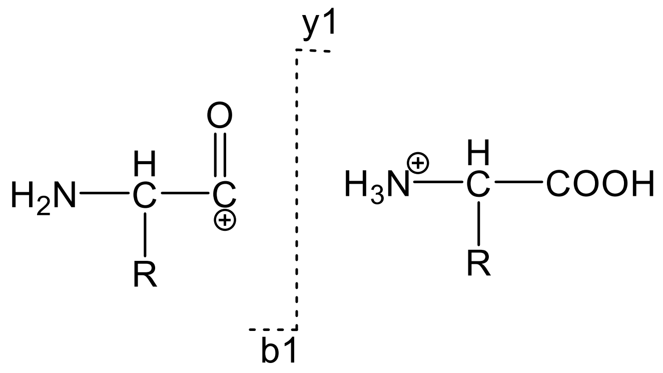

B

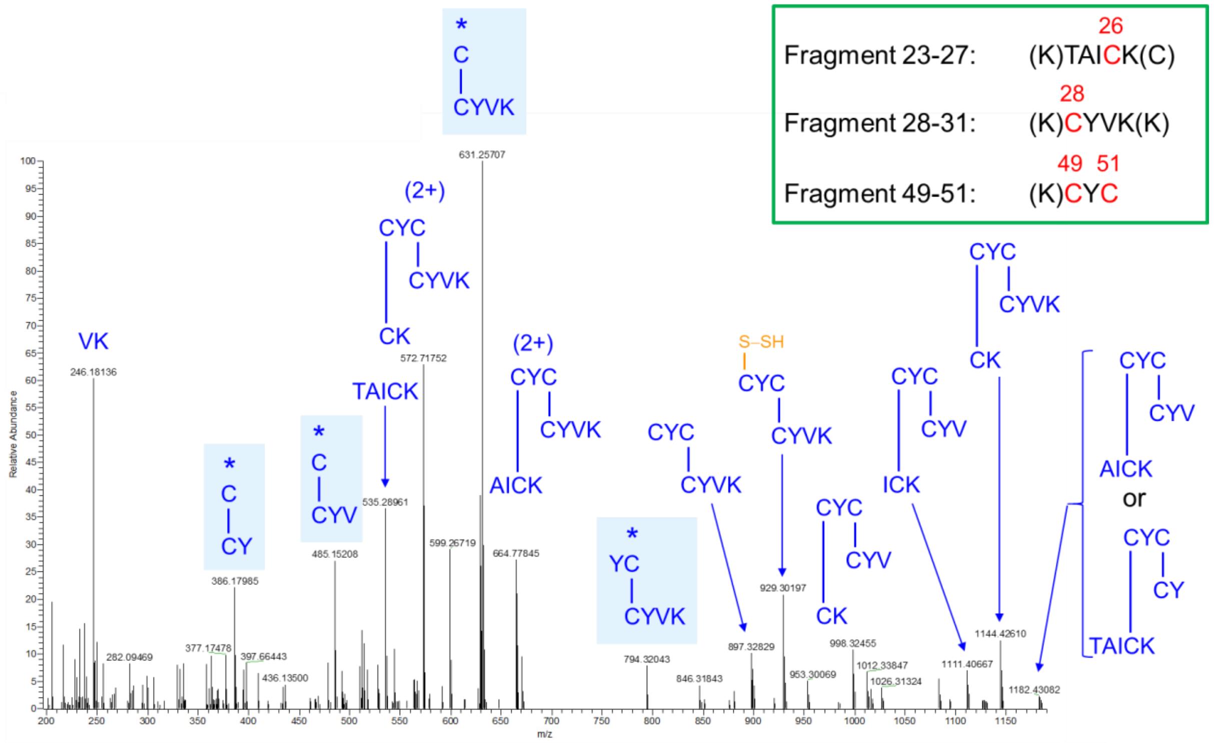

Figure S29. (A) Fragmentation of an amide bond in the higher-energy collisional dissociation (HCD) cell of MS. **(B)** MS/MS fragmentation spectrum of the 477.2042^{3+} *m/z* ion corresponding to the TAICK-CYC-CYVK triplet of peptide fragments at a collision energy of 27 eV. Ions marked with an asterisk and highlighted in light blue were crucial to determining the presence of disulfide bridges between Cys^{26} and Cys^{49}, and between Cys^{28} and Cys^{51}.

6. Reference

[1] G. Váradi, G. K. Tóth, Z. Kele, L. Galgóczy, Á. Fizil, G. Batta, Chemistry 2013, 19, 12684–12692.
